# Deciphering the role of *Th*HSF1 in the differential expression regulation of laccase isozymes in the white-rot fungus *Trametes hirsuta*

**DOI:** 10.1128/spectrum.01004-25

**Published:** 2025-11-25

**Authors:** Kun Wu, Rong Zhu, Shiwen Zhao, Chenkai Wang, Xinlei Zhang, Shenglong Liu, Zemin Fang, Yazhong Xiao, Juanjuan Liu

**Affiliations:** 1 School of Life Sciences and Medical Engineering, Anhui University 428675 , Hefei, Anhui, China; 2 Anhui Key Laboratory of Biocatalysis and Modern Biomanufacturing, Hefei, Anhui, China; 3 Anhui Provincial Engineering Technology Research Center of Microorganisms and Biocatalysis, Hefei, Anhui, China; University of Maryland Baltimore County, Baltimore, Maryland, USA

**Keywords:** heat shock transcription factor, laccase isozyme, copper stress, differential regulation, Hsp70

## Abstract

White-rot fungi exhibit responses to Cu^2+^, leading to a substantial increase in the production of certain laccase isozymes for industry utilization. Currently, studies on the differential expression mechanism of the laccase isozymes in white-rot fungi are limited. Three laccase isozymes, LacA, LacB, and LacF, were significantly induced in *Trametes hirsuta* AH28-2 when exposed to Cu^2+^, accompanied by a slight enhancement in the isozyme LacC level. Here, based on transcriptomics and proteomics, a nuclear-localized heat shock transcription factor, *Th*HSF1, was mined and exhibited as a copper-responsive protein involved in the differential regulation of laccase isozyme expression. A changed colony morphology and curved hyphal morphology were observed in three *Thhsf1*-silenced *T. hirsuta* AH28-2 transformants in response to Cu^2+^ stress. *Thhsf1* silencing resulted in downregulated transcriptional levels and activities of LacA, LacB, and LacF, but not LacC. EMSA assays further demonstrated the binding of *Th*HSF1 only to the promoter regions of *lacA*, *lacB,* and *lacF* containing HSE elements like CTTGAA. A previously reported Hsp70 homolog, *Th*HspA1, could interact with *Th*HSF1 and synergistically regulate the expressions of three laccase isozymes. *Th*HSF1 and *Th*HspA1 co-overexpression led to approximately 1.5-fold increased laccase activities, providing an efficient strategy to enhance laccase production.

**IMPORTANCE:**

White-rot fungi, especially *Trametes* species, are important producers of laccase. They typically express multiple laccase isozymes with distinct physicochemical properties in response to Cu^2+^. Elucidating the molecular mechanisms underlying differential laccase expression induced by Cu^2+^ is critical for enhancing laccase production through strain modification. This study demonstrates that *Th*HSF1 collaborates with *Th*HspA1 to regulate the expression of only three Cu^2+^-responsive laccase isozymes in *T. hirsuta* AH28-2. Co-overexpression of *Th*HSF1/*Th*HspA1 efficiently promotes laccase production. These findings also deepen our understanding of how white-rot fungi adapt to environmental Cu^2+^.

## INTRODUCTION

Laccase (benzenediol: oxygen oxidoreductases, EC 1.10.3.2) belongs to the family of copper-containing polyphenol oxidases. It serves as a polyphenol oxidase that oxidizes phenolic and aromatic compounds while simultaneously reducing molecular oxygen to water (1). Laccase plays diverse roles in different organisms, participating mainly in processes such as lignification, delignification, fungal morphogenesis, detoxification, plant infection, and oxidative stress (2–5). In the industry, it functions as a green catalyst and is utilized in various applications, including dye decolorization, detoxification of environmental pollutants, and pulp bleaching (6–8).

Laccases are widely distributed in fungi, plants, bacteria, lichens, and insects, particularly abundant in many white-rot fungi (9). Among them, basidiomycetes are the most efficient laccase producers (10), typically harboring multiple laccase isozyme genes in their genomes. These isozymes exhibit varied expression patterns in response to environmental factors, such as interfungal interactions, carbon and nitrogen source concentrations, as well as external inducing substances (metal ions and aromatic compounds from lignin monomer structures) (11). For metal ions, Cu^2+^ is considered the most effective inducer for enhancing laccase activity (12). Many basidiomycetes similarly show differential expression of multiple laccase isozymes under Cu^2+^ exposure, with examples including *Pleurotus ostreatus* (3 genes) (13), *Flammulina velutipes* (11 genes) (14), *Cerrena unicolor* (5 genes) (15), *Trametes hirsuta* 072 (5 genes) (16), *Ganoderma* sp. (6 genes) (17), and *Peniophora lycii* (18). These increment laccase isozymes usually have different properties and display distinct physiological features to not only allow fungi to succeed in a range of environments but also widen their industrial applications.

The prevailing viewpoint suggests that the induction and activation mechanism for fungal laccase transcription involves the interaction of specific transcription factors with upstream promoter regions in the presence of certain inducers (11). This hypothesis has been supported by the reported transcription factors, including Mac1, Ace1 and its homologs Cuf1, Ltf4, and *Th*SerRS controlling laccase expression upon exposure to Cu^2+^. In *Aspergillus fumigatus*, the *Af*Mac1-binding motif in *ctrC* is identified as 5′-TGTGCTCA-3′ (19). Ace1 interacts with the ACE element in laccase gene promoters, as observed in *Ceriporiopsis subvermispora* binding to the conserved motif (5′-CAGCGAAA-3′) within *lcs* and *Polyporus brumalis* ibrc05015 binding to the conserved motif (5‘-CAGCTCTAC-3′) within *Pblac1* (20, 21). Deletion of *cuf1* in *Cryptococcus neoformans* leads to a reduction in expression levels and activities of Lac1 (22). Furthermore, a yeast one-hybrid approach has been employed to identify transcription factors, including Ltf4 featuring an HTH DNA-binding domain and binding to DNA fragments containing the metal response element (MRE) within the *poxc* promoter region of *P. ostreatus* (23, 24) and *Th*SerRS showing a noncanonical activity of upregulating *lacA* transcription through binding to DNA fragments containing the xenobiotic response element (XRE) in *T. hirsuta* AH28-2 (25). These studies collectively highlight the complex and intricate mechanisms underlying laccase transcription during Cu^2+^ response. However, there is currently a lack of research focusing on elucidating the regulatory mechanisms responsible for the differential expression of fungal laccase isozymes mediated by specific transcription factors.

Heat shock transcription factors (HSFs) are highly conserved proteins across organisms ranging from fungi to humans. They serve as key regulators in multiple physiological processes, including heat stress response, oxidative stress tolerance, membrane lipid biosynthesis, hyphal growth, spore formation, and pathogenic fungal virulence (26–32). In stressful situations, HSFs undergo trimerization and bind to the heat shock elements (HSEs) located within the promoters of stress-inducible genes. This interaction triggers the activation of target proteins, such as molecular chaperones like heat shock protein 70 (Hsp70), along with other stress-related proteins (33, 34). Potential HSEs are highly prevalent within the upstream regulatory regions of numerous laccase genes in white-rot fungi (11). Additionally, since HSF can form a complex with DNA-binding transcription co-activator Ssa1 and interact with the promoter region of *lac1* in *C. neoformans* (35), suppression of *Pshsf1* results in a decline in laccase activity and diminished expressions of the isozymes *Ps*Lac4 and *Ps*Lac5 in *Phytophthora sojae* (29), and two spliced variants (*Tt*HSF2*α* and *Tt*HSF2*β*-I) of *Tt*HSF2 exhibit opposing effects on laccase expression in *Trametes trogii* (36), HSFs have been identified as crucial regulators governing laccase activity. However, it remains unknown whether HSF is involved in regulating all Cu^2+^-induced laccase isozymes within a species.

The white-rot fungus *T. hirsuta* AH28-2 was isolated from decaying wood in China (37). It possesses six laccase isozymes, with LacA, LacB, and LacC being overexpressed during Cu^2+^ exposure, as reported previously (38, 39). Recently, a new Cu^2+^-induced isozyme, LacF, was also characterized (40). Here, a nuclear-localized copper-responsive heat shock transcription factor, *Th*HSF1, was identified through comparative proteomic and transcriptomic analyses of *T. hirsuta* AH28-2 mycelia treated with Cu^2+^. The gene silencing experiments, quantitative reverse transcription-PCR (qRT-PCR) analysis, electrophoretic mobility shift assay (EMSA), and fluorescence polarization (FP) collectively demonstrated specific and direct regulation of *Th*HSF1 on *lacA*, *lacB*, and *lacF* while excluding *lacC*. The DNA-binding transcription factor *Th*HspA1, previously reported to function in LacA regulation upon aromatic stress (41), was also targeted by *Th*HSF1 during Cu^2+^ induction. *Th*HSF1 and *Th*HspA1 could interact to form a complex and synergistically enhance the expressions of LacA, LacB, and LacF. Co-overexpression of *Th*HSF1/*Th*HspA1 provided an efficient strategy for boosting laccase production.

## RESULTS

### Overview of comparative proteomic and transcriptomic analyses of *T. hirsuta* AH28-2 exposed to Cu^2+^

Cu^2+^ is an efficient inducer for the overexpression of laccase isozymes in *T. hirsuta* AH28-2, such as LacA, LacB, LacC, and LacF (39, 40). To investigate the regulatory mechanism of these laccases, *T. hirsuta* AH28-2 was first inoculated into liquid XH media supplemented with varying concentrations of CuSO_4_ (100–1,000 µM) to determine the optimal induction conditions. The highest laccase activity was observed at 100 µM Cu^2+^ (Fig. S1A and B). qRT-PCR analysis further revealed a significant increase in transcriptional levels of these four isozymes during the first 48 h of 100 µM Cu^2+^ treatment (Fig. S1C).

**Fig 1:**
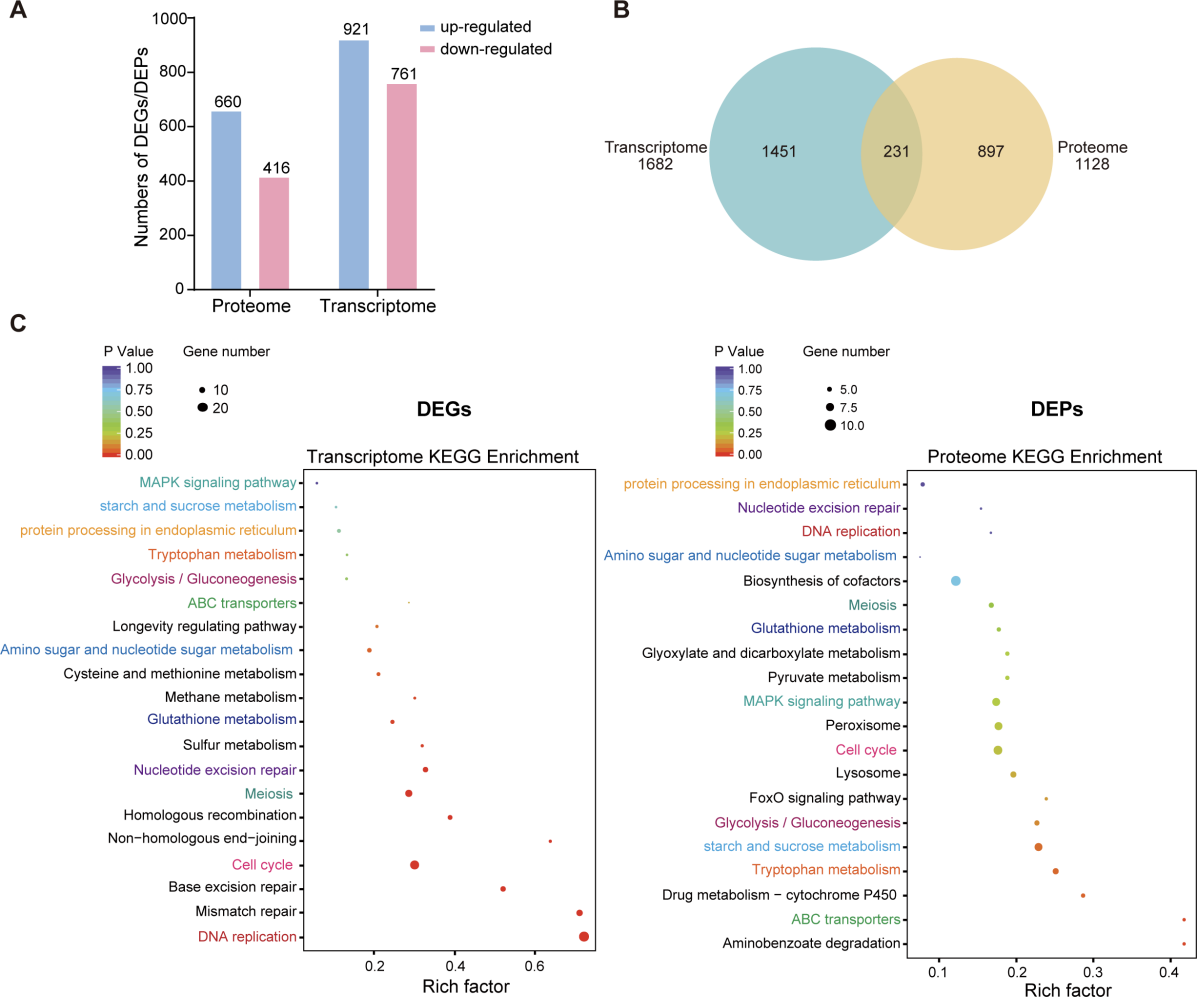
Transcriptomic and proteomic analyses of *T. hirsuta* AH28-2 upon exposure to 100 µM Cu^2+^ for 48 h. (**A**) Numbers of the upregulated and downregulated differentially expressed genes (DEGs) and differentially expressed proteins (DEPs). (**B**) Venn diagrams. (**C**) Kyoto Encyclopedia of Genes and Genomes (KEGG) pathway enrichment analysis of DEGs (left panel) and DEPs (right panel) identified in *T. hirsuta* AH28-2 after 48 h of 100 µM Cu²^+^ exposure. Each dot represents an enriched KEGG pathway. The X-axis indicates the rich factor (the ratio of the number of DEGs/DEPs in a pathway to the total number of genes annotated in that pathway), the size of the dot indicates the number of genes/proteins involved, and the color reflects the adjusted *P* value. KEGG pathways that are enriched in both omics data sets are labeled with the same font color on the Y-axis to facilitate comparison.

Laccase expression in most fungi is regulated strictly at the transcriptional level. The mycelia, exposed to 100 µM Cu^2+^ for 48 h, were collected for proteomic and transcriptomic analyses to identify Cu²^+^-responsive transcription factors or co-regulators. A total of 4,499 proteins and 10,984 genes were detected, of which 1,128 proteins (660 upregulated and 416 downregulated) and 1,682 genes (921 upregulated and 761 downregulated) were identified as differentially expressed genes (DEGs)/differentially expressed proteins (DEPs) (*P* value < 0.05), respectively (Fig. 1A). After overlapped analysis, 231 DEGs/DEPs were consistent between both omics (Fig. 1B). To evaluate the significance of this overlap against a null model, the DEG and DEP sets were treated as independent samples drawn from all detected transcripts, and a hypergeometric test was applied. The expected overlap under the assumption of independence was 173, whereas the observed overlap of 231 was significantly greater. Both upregulated and downregulated features were included in the overlap analysis, and the 231 DEG–DEP pairs were further stratified by expression directionality, with concordant pairs predominating. A complete list of these pairs, including fold changes and expression directions, is provided in Table S1.

According to Gene Ontology (GO) analysis, DEGs/DEPs associated with cellular processes and metabolic processes in biological processes; binding, oxidoreductase, and catalytic activities in molecular functions; and membrane and nucleus in cell components were increased (Fig. S2). The Kyoto Encyclopedia of Genes and Genomes (KEGG) analysis revealed enrichment of pathways such as biosynthesis of cofactors, peroxisome, glutathione metabolism, and ABC transporters, all of which are closely related to metal ion homeostasis (Fig. 1C), indicating that *T. hirsuta* AH28-2 cells can uptake Cu^2+^ and incorporate Cu²^+^ into cellular processes, in accordance with our recent study and other reports (40, 42, 43). Furthermore, similarly to the response to different concentrations of Cu²^+^ (40), 100 µM Cu²^+^ exposure led to notable increases in the expression levels of many antioxidant enzymes, including glutathione peroxidase (GPX), glutathione transferase (GST), catalase (CAT), superoxide dismutase (SOD), laccases, and some cytochrome P450s (CYP450s) (Fig. S3).

A total of 26 differentially expressed genes/proteins (*P* value < 0.05) were identified as transcription factors (TFs) potentially involved in the response to Cu^2+^ and laccase expression (Table 1). They encompassed a diverse range of types, including zinc finger proteins (CCHC-type, GATA-type, Zn_2_Cys_6_-type, and C_2_H_2_-type), bHLH, bromodomain, winged helix repressor, homeodomain-like, heat shock, lambda repressor-like, and HMG. These findings suggest that Cu²^+^ may function not only as a cofactor but also as a regulator of global transcriptional responses, thereby contributing to the maintenance of cellular homeostasis. Notably, among the 17 upregulated DEPs, an HSF homolog, GME4084, exhibited a 3.6-fold increase in protein abundance upon Cu²^+^ exposure, ranking second among all DEPs—immediately after GME4300 (*Th*8421), which has been demonstrated to play a critical role in stress defense mechanisms in *T. hirsuta* AH28-2 (44)—thereby underscoring its potential significance in the cellular response to Cu²^+^. Together with the observed response stress pattern and the documented role of HSF in responding to Cu^2+^ in white-rot fungi (36), GME4084 may act as a central regulator linking metal sensing to downstream gene expression, including laccase regulation.

**TABLE 1:** The transcription factors with significant differential expression in both the transcriptome and proteome in response to Cu^2+^

ID	TF name	Fold change (DEGs)	*P* value (DEGs)	Fold change (DEPs)	*P* value (DEPs)
GME10088_g	Zinc finger, CCHC-type	2.107908998	7.51263E-07	1.57532043	0.045406063
GME2510_g	GATA type zinc finger	1.5869992	0.001778069	1.575952684	0.033066179
GME4340_g	GATA type zinc finger	1.795357064	8.26901E-05	1.606574751	0.0050983
GME8357_g	C_2_H_2_ zinc finger	1.838851047	0.000046861	1.699217276	0.038145886
GME2432_g	Nucleic acid-binding	4.158711649	3.68075E-06	1.792502092	0.048178675
GME11621_g	bHLH	2.019026652	0.000276094	1.815055236	0.038209986
GME1888_g	Nucleic acid-binding	3.018344695	0.003428255	1.891711089	0.007754755
GME1535_g	Zn_2_Cys_6_	1.942188908	1.84772E-05	1.927006421	0.009949426
GME1466_g	Zn_2_Cys_6_	1.663835455	0.000561435	2.175673245	0.007131924
GME4300_g	Zn_2_Cys_6_	3.446857579	0.000112759	3.581676361	0.042828236
GME11612_g	Bromodomain	1.401473113	0.026117111	2.381173829	0.00791596
GME1868_g	C_2_H_2_ zinc finger	1.874052153	2.17393E-05	2.521320205	0.018508829
GME6110_g	Nucleic acid-binding	3.010867141	5.3032E-10	2.634908185	0.017968958
GME10687_g	GATA type zinc finger	1.808039087	0.000046184	2.801842872	0.000187236
GME5442_g	Winged helix repressor	3.381799047	0.001132513	2.985381372	0.034732672
GME1417_g	Homeodomain-like	15.39403547	5.17963E-17	89.33758714	0.012148507
GME4084_g	Heat shock	1.406437642	0.019737603	3.578007108	0.04777482
GME2780_g	Lambda repressor-like	0.356524313	0.002240857	0.31439301	0.025876009
GME2836_g	HMG	0.559144061	0.000079104	0.32075343	0.012164611
GME282_g	Nucleic acid-binding	0.733516994	0.036540703	0.355175569	0.013484173
GME5687_g	Nucleic acid-binding	0.687861656	0.015040272	0.462004093	0.01563177
GME7650_g	Nucleic acid-binding	0.450292964	5.50343E-08	0.556160881	0.001219601
GME851_g	Nucleic acid-binding	0.475954308	1.37765E-06	0.600757576	0.005478666
GME351_g	Nucleic acid-binding	0.727044028	0.03396102	0.630561742	0.00850075
GME1542_g	C_2_H_2_ zinc finger	0.270782077	3.85336E-18	0.674106415	0.030503107
GME7020_g	HMG	0.464816591	4.25848E-07	0.767384294	0.014950677

### GME4084 is a nuclear-localized HSF with DNA-binding capacity and transcriptional activation activity responding to Cu^2+^ exposure

**Fig 2:**
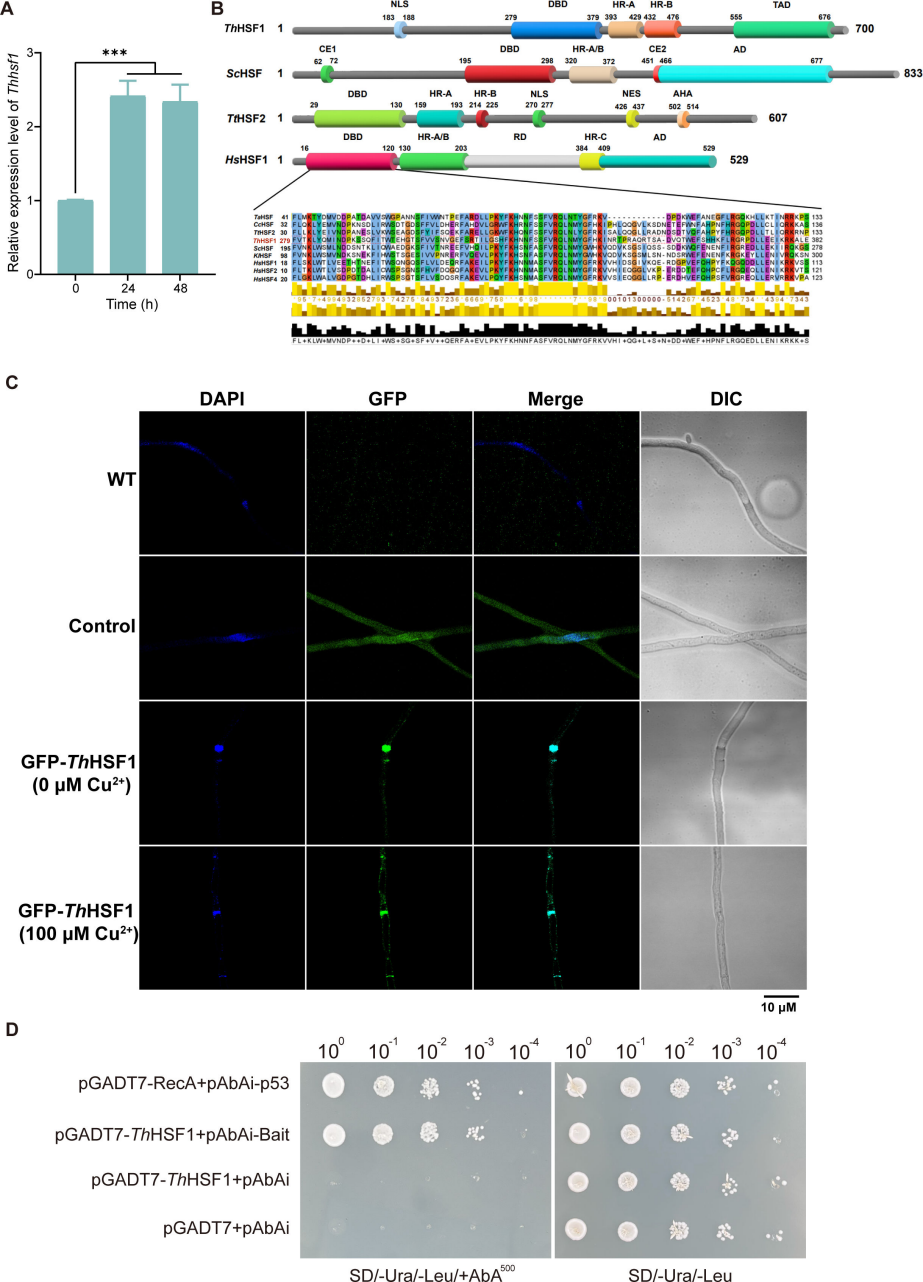
A nuclear-localized transcription factor *Th*HSF1 exhibits upregulation in *T. hirsuta* AH28-2 in response to Cu^2＋^. (**A**) The transcriptional levels of *Th*HSF1 increased following copper exposure. Data show mean ± SD, *n* = 3. ***P < 0.001. (**B**) The amino acid alignment and structural prediction of *Th*HSF1 are compared to homologs from six other species. *Ta*HSF (Gene ID: 123114421) of *T. aestivum L*., *Cc*HSF (Gene ID: 6017888) of *C. cinerea*, *Tt*HSF2 of *T. trogii*, *Sc*HSF1 (GenBank: CAA96777.1) of *S. cerevisiae*, *Kl*HSF (GenBank: CAA38950.1) of *Kluyveromyces lactis*, and *Hs*HSF1 (Gene ID: 3297), *Hs*HSF2 (GenBank: AAA36017.1), and *Hs*HSF4 (GenBank: BAA84581.1) of *H. sapiens* are concluded. (**C**) GFP-*Th*HSF1 was localized in the nucleus. The WT strains, control transformants (EGFP overexpression), and *gfp-Thhsf1* overexpressed *T. hirsuta* AH28-2 transformants were grown on microscope slides with XH agar medium, fixed with cold methanol, and then stained with DAPI. GFP-*Th*HSF1 is shown in green, and DAPI staining of the nucleus is shown in blue. (**D**) *Th*HSF1 specifically bound to *lacA* promoter in yeast. Experiments were repeated at least three times, and representative results are shown. Scale bars = 10 µm.

The genome of *T. hirsuta* AH28-2 harbored four putative heat shock-type TF genes. Among them, only one gene (*GME4084*, designated as *Thhsf1*) exhibited significant and consistent upregulation during the initial 48 h of Cu^2+^exposure, showing a 1.4-fold increase in transcript levels as determined by RNA-seq and a 2.3-fold increase confirmed by qRT-PCR validation (Table 1; Fig. 2A; Fig. S4). This gene encoded a 700-amino acid protein. BLASTP analysis in the NCBI database showed that the closest homolog of *Th*HSF1 was an HSF-type DNA-binding domain-containing protein from *Trametes elegans* (Sequence ID: KAI0765634.1), with a sequence identity of 87%. This indicates that *Th*HSF1 is a conserved HSF-like TF among white-rot fungi. Multiple sequence alignment revealed that the DNA-binding domain (DBD) of *Th*HSF1 shares 50%, 48.57%, 44.76%, and 45.13% sequence identity with the DBDs of *T. trogii Tt*HSF2 (36), *Saccharomyces cerevisiae* (GeneBank: CAA96777.1), *Triticum aestivum* (Gene ID: 123114421), and *Homo sapiens* (Gene ID: 3297) homologs, respectively (Fig. 2B; Fig. S5). Structural modeling, based on templates from *T. trogii* HSF2 (36), *H. sapiens* HSF1 (45), and *S. cerevisiae* HSF1 (46) indicated that *Th*HSF1 is predicted to contain three additional characteristic domains: HR-A (393 to 429 aa), HR-B (432 to 476 aa), and a C-terminal transactivation domain (TAD) (555 to 676 aa). The nuclear localization signal (NLS) of *Th*HSF1, predicted using the NucPred website (47), is located at aa position 183–188 (PRKRSY).

The overexpression vector of *Th*HSF1-GFP was constructed by fusing *egfp* to the full-length cDNA of *Th*HSF1 at the C-terminus and under the control of *the Agaricus bisporus* promoter *gapdhII* and transformed into *T. hirsuta* AH28-2 to investigate the subcellular localization of *Th*HSF1. The positive transformants were confirmed by genomic validation and fluorescence screening. Transformants with only EGFP overexpression were used as controls. Intense GFP fluorescence was predominantly accumulated in the nucleus compared to the wild-type (WT) strain and control transformants (Fig. 2C). Hence, *Th*HSF1 is mainly a nuclear protein.

Using the *lacA* promoter as an example, the DNA-binding capacity of *Th*HSF1 was investigated in *S. cerevisiae* via the yeast one-hybrid system (25). A 150-bp fragment of the *lacA* promoter (−1,800 to −1,650 bp) was cloned upstream of the aureobasidin A (AbA) resistance gene *AUR1-C* in the pAbAi vector and served as the bait DNA. Yeast transformants co-expressing the full-length cDNA of *Th*HSF1 were able to grow on SD/-Ura/-Leu/AbA^500^ plates (Fig. 2D), indicating specific binding activity. Furthermore, the transcriptional activation activity of *Th*HSF1 was verified by its ability to activate the *lacZ* reporter gene upon co-transformation with the modified pRS414 plasmid (48) carrying the full-length *lacA* promoter and the *Escherichia coli lacZ* coding sequence. The *β*-galactosidase (LacZ) activity in the pGADT7-*Th*HSF1 plasmid co-transformation group was 17.6 ± 3.0 U/mL, which was significantly higher than that of the empty pGADT7 vector control group (3.2 ± 1.3 U/mL; *P* < 0.001).

### Silencing *Thhsf1* in*T. hirsuta* AH28-2 leads to heightened sensitivity toward elevated temperatures and increased Cu^2+^ stress

**Fig 3:**
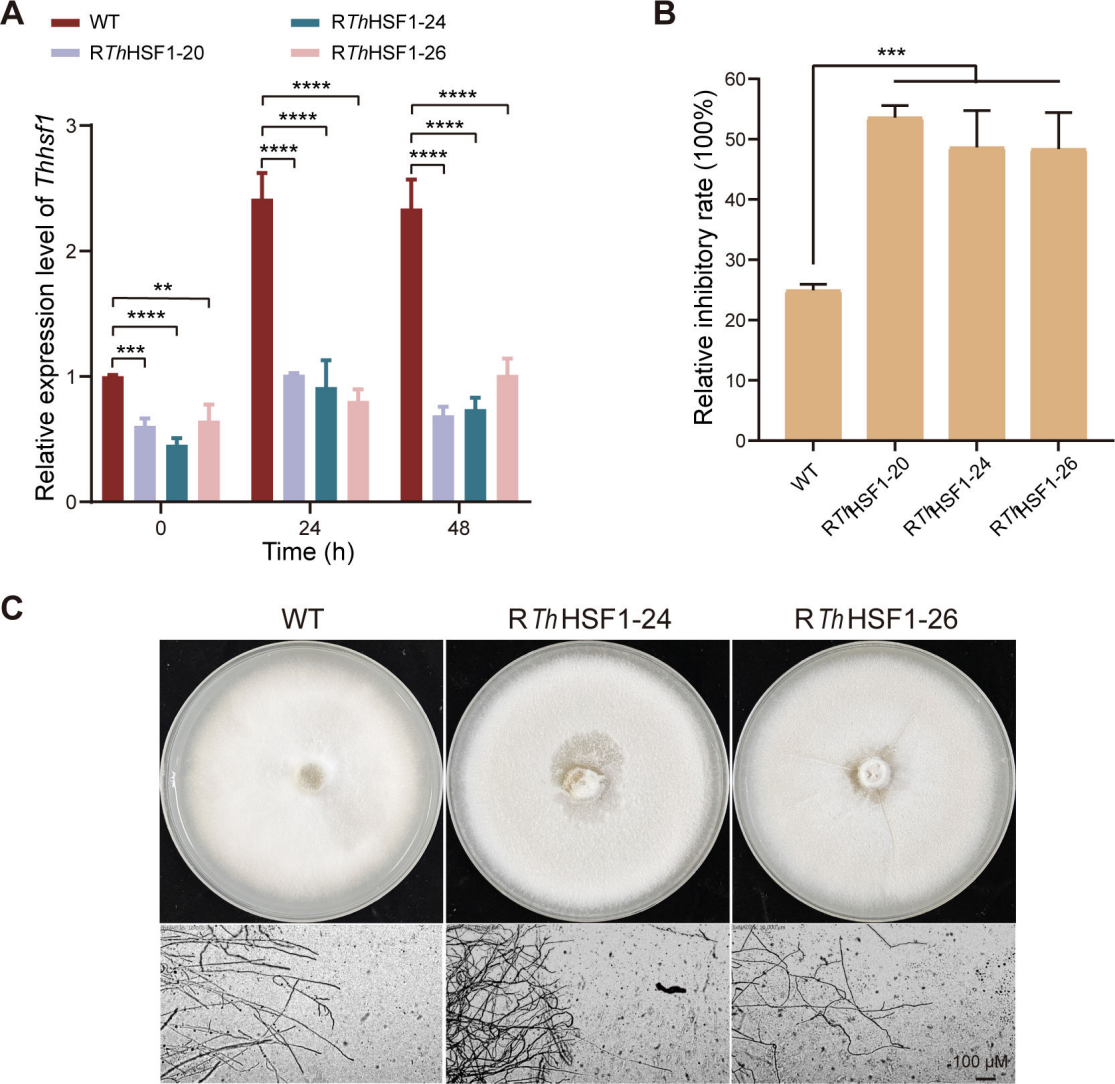
Silencing *Thhsf1* in *T. hirsuta* AH28-2 leads to heightened sensitivity toward elevated temperatures and increased Cu^2+^ stress. (**A**) *Thhsf1* expression was downregulated in three randomly chosen transformants. The WT and *Thhsf1*-silenced *T. hirsuta* AH28-2 were cultivated in liquid XH medium supplemented with 100 µM CuSO_4_, harvested every 24 h, and subjected to RNA extraction. The transcriptional level of *Thhsf1* in WT at 0 h of induction was set as the baseline. (**B**) The inhibition rates of strains under an elevated temperature of 37°C. The colony diameters were measured and subjected to statistical analysis. Inhibitory rate = (the diameter of 28°C-cultured strain – the diameter of 37°C-cultured strain)/(the diameter of 28°C-cultured strain) ×100%. (**C**) The *Th*HSF1-silenced strains showed increased sensitivity to Cu²^+^ stress compared to the WT. Growth of strains on XH agar plates supplemented with 100 µM Cu²^+^ is shown. Experiments were repeated at least three times, and representative results are shown. Data show mean ± SD, *n* = 3. **P < 0.01, ***P < 0.001, and *P* < 0.0001.

The *Thhsf1*-silenced transformants of *T. hirsuta* AH28-2 were generated to evaluate the role of *Th*HSF1 during Cu^2+^ exposure. A total of 33 *Thhsf1*-silenced transformants were obtained, as confirmed by genomic PCR and qRT-PCR analysis (Fig. S6). Three randomly selected transformants, namely, R*Th*HSF1-20, R*Th*HSF1-24, and R*Th*HSF1-26, exhibited a significant reduction in *Thhsf1* transcripts, ranging from 57% to 71% compared to the WT strain after exposure to Cu^2+^ for 48 h. (*P* < 0.0001; Fig. 3A).

The functional validation of *Th*HSF1 *in vivo* was initially conducted by exposing these strains to an elevated temperature of 37°C. In contrast to the phenotype observed during normal growth at 28°C for 7 d, the mycelial growth rate of three *Thhsf1*-silenced transformants showed greater reductions (48%–53%) than that of the WT strain (25%) (Fig. 3B; Fig. S7), indicating a significant role of *Th*HSF1 in thermal stress adaptation. In contrast, silencing of *Th*HSF1 did not exhibit a significant impact on fungal growth under the other stress conditions tested. Exposure to H₂O₂, *o*-toluidine, or Congo Red did not lead to more pronounced growth inhibition in *Th*HSF1-silenced strains compared to the wild-type strain, and no substantial differences in colony size or morphology were observed under these conditions, suggesting that the growth defect caused by *Th*HSF1 silencing is not generally amplified by unrelated stressors and that *Th*HSF1 is unlikely to function as a general stress response regulator (Fig. S7). However, cultivation of these strains on XH agar plates containing 100 µM Cu^2+^ revealed a changed colony morphology and a curved hyphal morphology among *Thhsf1*-silenced transformants (Fig. 3C). These findings suggest that *Th*HSF1 plays a role not only in defense against high-temperature stress but also in response to Cu^2+^ stress in *T. hirsuta* AH28-2.

### *Th*HSF1 regulates the expressions of LacA, LacB, and LacF, but not LacC

**Fig 4:**
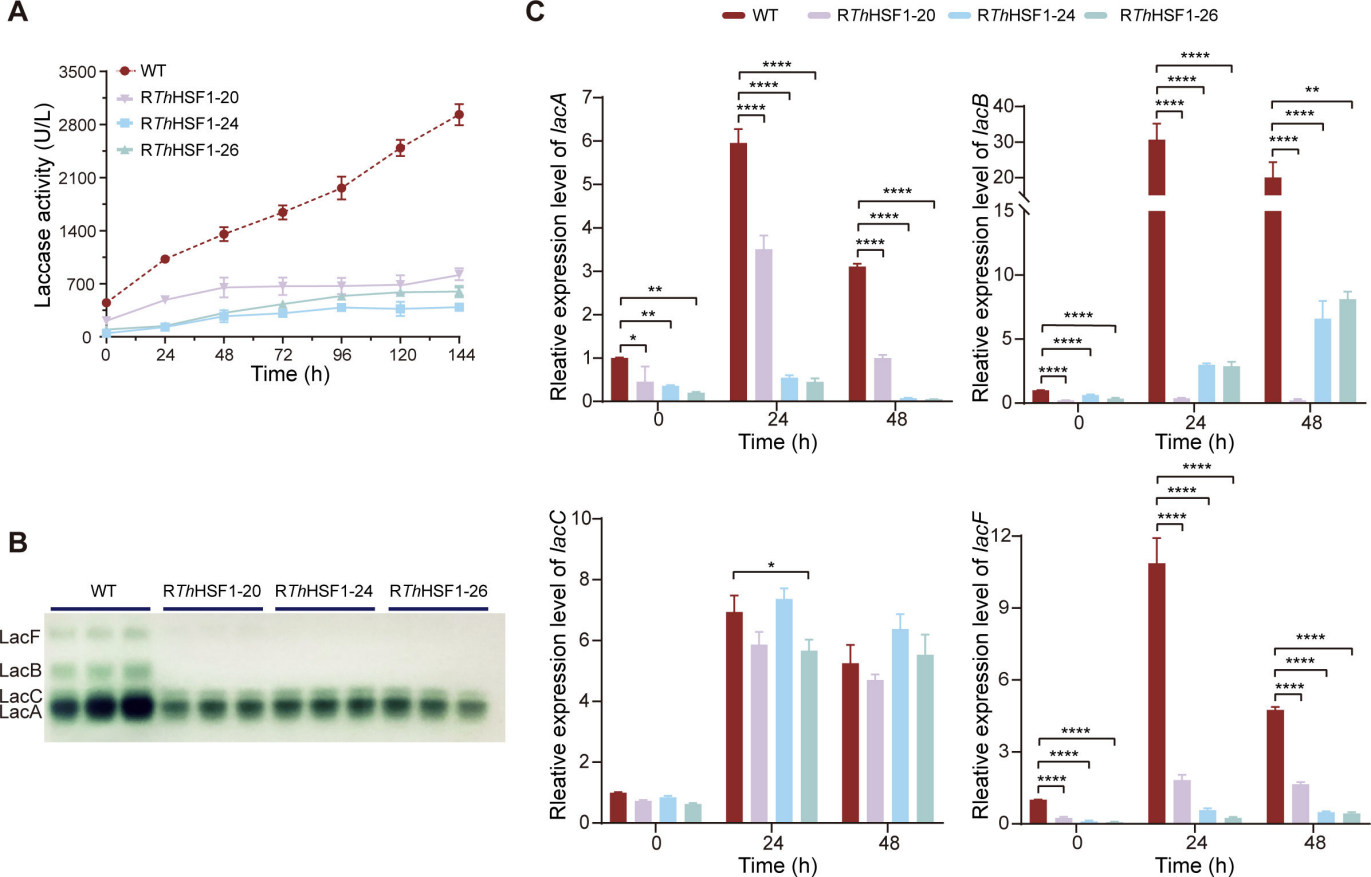
*Th*HSF1 positively regulates the expressions of LacA, LacB, and LacF in response to Cu^2+^, except for LacC. The total laccase activity (**A**), laccase isozymes detected by native-PAGE (**B**), and the transcriptional levels of laccase isozymes (**C**) in WT and *Thhsf1*-silenced *T. hirsuta* AH28-2 are shown. Strains were cultivated in liquid XH medium supplemented with 100 µM CuSO_4_, harvested every 24 h, centrifuged, and subjected to activity detection and RNA extraction. The transcriptional levels of each gene in the WT strain at 0 h of induction were set as the baseline in each subgraph of **C**. Experiments were repeated at least three times, and representative results are shown. Data show mean ± SD, *n* = 3. *P < 0.05, **P < 0.01, and ****P < 0.0001.

The laccase activity and expression profile were measured in WT and *Thhsf1*-silenced strains. As shown in Fig. 4A, the total laccase activity decreased from 2,900 U/L to a range of 390–820 U/L upon Cu^2+^ induction after silencing *Thhsf1*. Native-PAGE analysis revealed a notable reduction in the expressions of LacA, LacB, and LacF in these silenced transformants, while the expression of LacC remained unchanged (Fig. 4B). qRT-PCR results further supported the isozyme expression profile as the transcription levels of *lacA*, *lacB*, and *lacF* were much lower in *Thhsf1*-silenced transformants compared to the WT strain (Fig. 4C). For instance, *Thhsf1* silencing led to a reduction in *lacA* transcripts by 3.1- to 61.7-fold, *lacB* transcripts by 2.4- to 93.7-fold, and *lacF* transcripts by 2.8- to 10.9-fold (*P* < 0.01 or *P* < 0.0001). In contrast, *Thhsf1* silencing resulted in no noticeable change in the transcription levels of *lacC*. These results indicated that *Th*HSF1 functioned in the differential regulation of laccase isozyme transcription.

### *Th*HSF1 directly binds to the *lacA*, *lacB,* and *lacF* promoters

**Fig 5:**
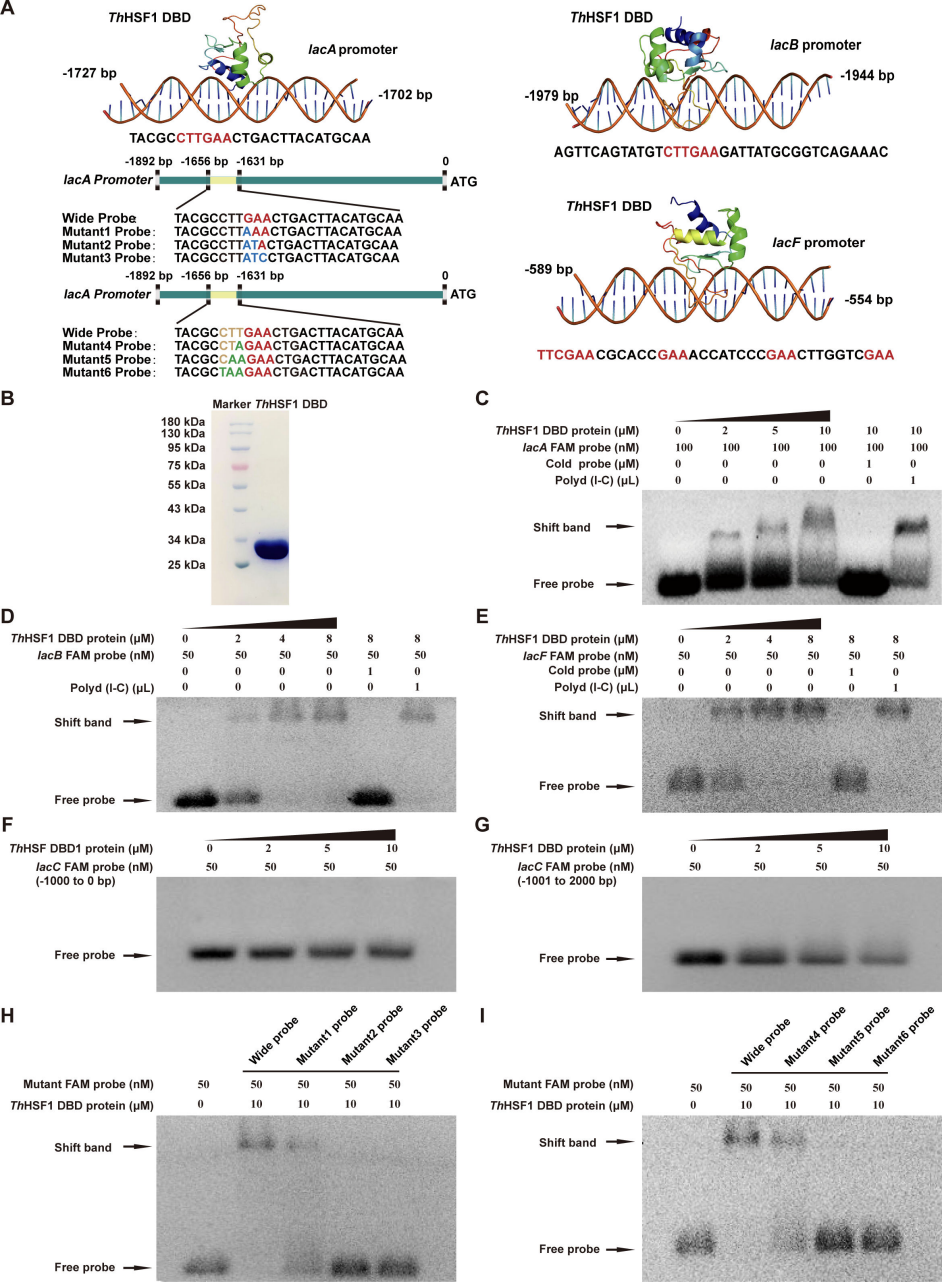
*Th*HSF1 binds directly to the promoter regions of *lacA*, *lacB*, and *lacF*. (**A**) Structure modeling of *Th*HSF1 interacting with *lacA*, *lacB,* and *lacF* promoters using HDOCK software. The simulated DNA fragments involved in interaction are indicated below each model. The probe sequences derived from the *lacA* promoter used in EMSA were labeled with pre-mutation sequences highlighted in red and brown, while post-mutation sequences were indicated by blue and green. (**B**) The *Th*HSF1 DBD was heterologously expressed in *E. coli* BL21 and purified. (**C–E**) EMSA assays showed that *Th*HSF1 directly bound to the *lacA* promoter (−1,727 to −1,702 bp) (**C**), the *lacB* promoter (–1,979 to –1,944 bp) (**D**), and the *lacF* promoter (–589 to –554 bp) (**E**). Lane assignments: lane 1, probe only; lanes 2–4, probe adding with increasing concentrations of *Th*HSF1 DBD; lane 5, probe adding with *Th*HSF1 DBD and speciﬁc competitor; lane 6, probe adding with *Th*HSF1 DBD and non-speciﬁc competitor. Poly d(I-C) was added as the non-speciﬁc competitor, and a 10-fold identical unlabeled DNA probe was added as the speciﬁc competitor. (**F, G**) EMSA assays exhibited no interaction between *Th*HSF1 and the *lacC* promoter. The upstream 2,000-bp promoter region of *lacC* was divided into two segments, and primers labeled with FAM were used to amplify probes of the first 1,000 bp (**F**) and the last 1,000 bp (**G**). (**H, I**) EMSA assays of DNA binding of *Th*HSF1 to mutant probes. Experiments were repeated at least three times, and representative results are shown.

HDOCK was employed to simulate the three-dimensional (3D) models of either the full-length or DBD of *Th*HSF1 and the promoters of laccase isozymes (*lacA*, *lacB*, and *lacF*). *Th*HSF1 DBD exhibited a binding affinity for the DNA fragment containing the conserved CTTGAA sequence in both the *lacA* promoter (−1,727 to −1,702 bp) and *lacB* promoter (−1,967 to −1,962 bp) (Fig. 5A; Fig. S8). In comparison, *Th*HSF1 DBD interacted with the *lacF* promoter region (−589 to −554 bp), which contains conserved nGAAn sequences.

EMSA and FP assays were further performed to investigate these interactions *in vitro*. The *Th*HSF1 DBD was heterologously expressed in *E. coli* and purified (Fig. 5B). Three DNA fragments, each containing the binding regions, were labeled with 6-carboxyfluorescein (6-FAM) and used as probes. *Th*HSF1 was observed to form a chemiluminescent complex with each probe. The poly d(I-C) had no effect on the shifted band, while the addition of a 10-fold excess of nonlabeled DNA fragment hindered complex formation (Fig. 5C through E). FP assays revealed dissociation constants (Kd) of approximately 961 nM, 5,464 nM, and 4,826 nM for *Th*HSF1 binding to *lacA*, *lacB*, and *lacF* DNA probes, respectively (Fig. S9). In contrast, validation of the two 1,000-bp probes, designed from *lacC* promoter regions and amplified with FAM-labeled primers, showed no binding to *Th*HSF1 (Fig. 5F and G).

The 26-bp *lacA* probe was chosen for mutagenesis to validate the specific binding site in docking models. Both EMSA and FP results conclusively indicated that the core conserved sequence CTTGAA within the *lacA* promoter region was crucial for *Th*HSF1 binding (Fig. 5H and I; Fig. S8). Specifically, the binding site in the *lacB* promoter also exhibited the same conserved motif CTTGAA as *lacA*. These findings suggested that *Th*HSF1 differentially regulated the expressions of laccase isozymes by directly interacting with their respective promoter regions.

### Co-overexpression of *Th*HSF1 and *Th*HspA1 in *T. hirsuta* AH28-2 significantly enhances the expressions of LacA, LacB, and LacF

**Fig 6:**
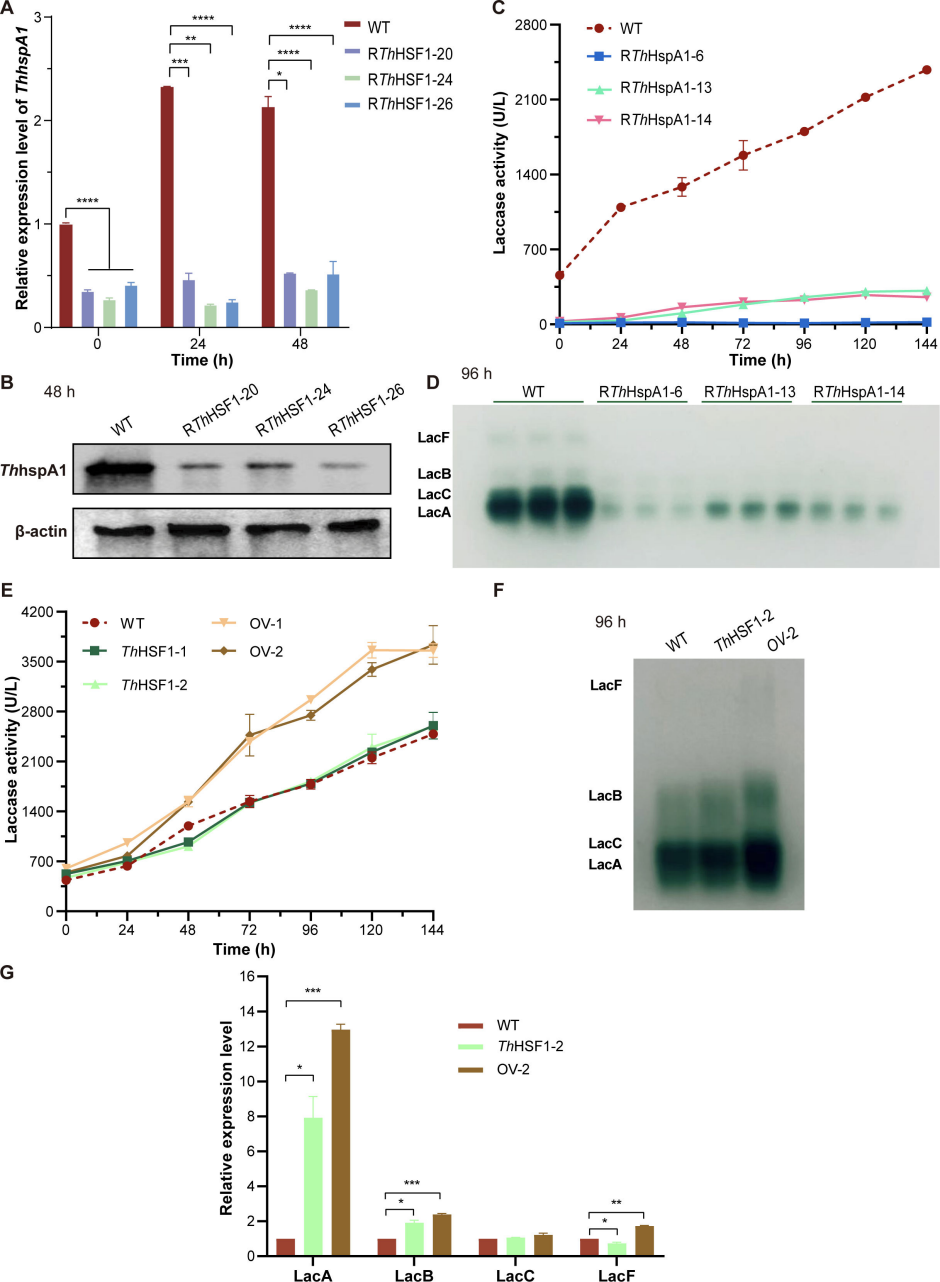
The cooperation of *Th*HSF1 and *Th*HspA1 is involved in laccase isozyme regulation in *T. hirsuta* AH28-2 upon Cu^2+^ induction. (**A, B**) The transcriptional level (**A**) and expression of *ThhspA1* (**B**) were downregulated after silencing *Thhsf1*. The transcriptional level of *ThhspA1* in the WT strain at 0 h of induction was set as the baseline. (**C, D**) The total laccase activity (**C**) and expression of four laccase isozymes (**D**) exhibited a reduction in *ThhspA1*-silenced transformants compared to the WT strain in response to Cu^2+^. (**E–G**) Co-overexpression of *Th*HSF1/*Th*HspA1 in *T. hirsuta* AH28-2 enhanced the total laccase activity (**E**), expressional levels (**F**), and transcriptional levels (**G**) of LacA, LacB, and LacF. *Th*HSF1 overexpression showed no significant effect. The transcriptional levels of each gene in the WT strain at 24 h of induction were set as the baseline in **G**. Experiments were repeated at least three times, and representative results are shown. Data show mean ± SD, *n* = 3. *P < 0.05, **P < 0.01, ***P < 0.001, and *P* < 0.0001.

The expression profile of *Th*HspA1, a potent downstream target of HSF (49) and known to be involved in *lacA* transcription upon *o*-toluidine induction in *T. hirsuta* AH28-2 (41), was analyzed. After 24 h of Cu^2+^ treatment, *ThhspA1* transcripts decreased by 79%, 90%, and 89% in *Thhsf1*-silenced transformants compared to the WT strain (Fig. 6A). Western blot analysis showed a significant reduction in *Th*HspA1 expression after silencing *Thhsf1* (Fig. 6B), demonstrating a positive regulatory role of *Th*HSF1 on *ThhspA1*. Furthermore, *Th*HspA1 regulated the expressions of the four Cu^2+^-mediated laccase isozymes as the activities of LacA, LacB, LacC, and LacF were all significantly lower in three *ThhspA1-*silenced transformants (41) compared to the WT strain (Fig. 6C and D, *P* < 0.01 or *P* < 0.0001).

*Th*HSF1 overexpression and *Th*HSF1/*Th*HspA1 co-overexpression were performed in *T. hirsuta* AH28-2 to study the cooperation of *Th*HSF1 and *Th*HspA1 in regulating laccase isozyme activity. The recombinant transformants were chosen for flask cultivation to detect the laccase production. The total laccase activities of both randomly chosen *Thhsf1-*overexpressing transformants, namely, *Th*HSF1-1 and *Th*HSF1-2, were comparable to or slightly higher than those of the WT strain throughout the entire culture period, reaching approximately 2,500 U/L at 144 h. However, the co-transformants OV-1 and OV-2 exhibited significantly higher laccase activity, with levels reaching about 3,700 U/L at 144 h (Fig. 6E). The biomass of these strains was similar. Native-PAGE and qRT-PCR analyses were further conducted to compare the WT, *Th*HSF1-2, and OV-2 strains. The results indicated a significant increase in the expression and transcriptional levels of LacA, LacB, and LacF in OV-2 compared to WT and *Th*HSF1-2 (Fig. 6F and G; *P* < 0.05, *P* < 0.01, or *P* < 0.0001). However, overexpression of *Th*HSF1 or co-overexpression of *Th*HSF1/*Th*HspA1 almost did not affect LacC. Thus, *Th*HSF1 collaborated with *Th*HspA1 to enhance the expressions of LacA, LacB, and LacF in *T. hirsuta* AH28-2.

### *Th*HSF1 forms a regulatory complex with *Th*HspA1 to function in laccase transcription

**Fig 7:**
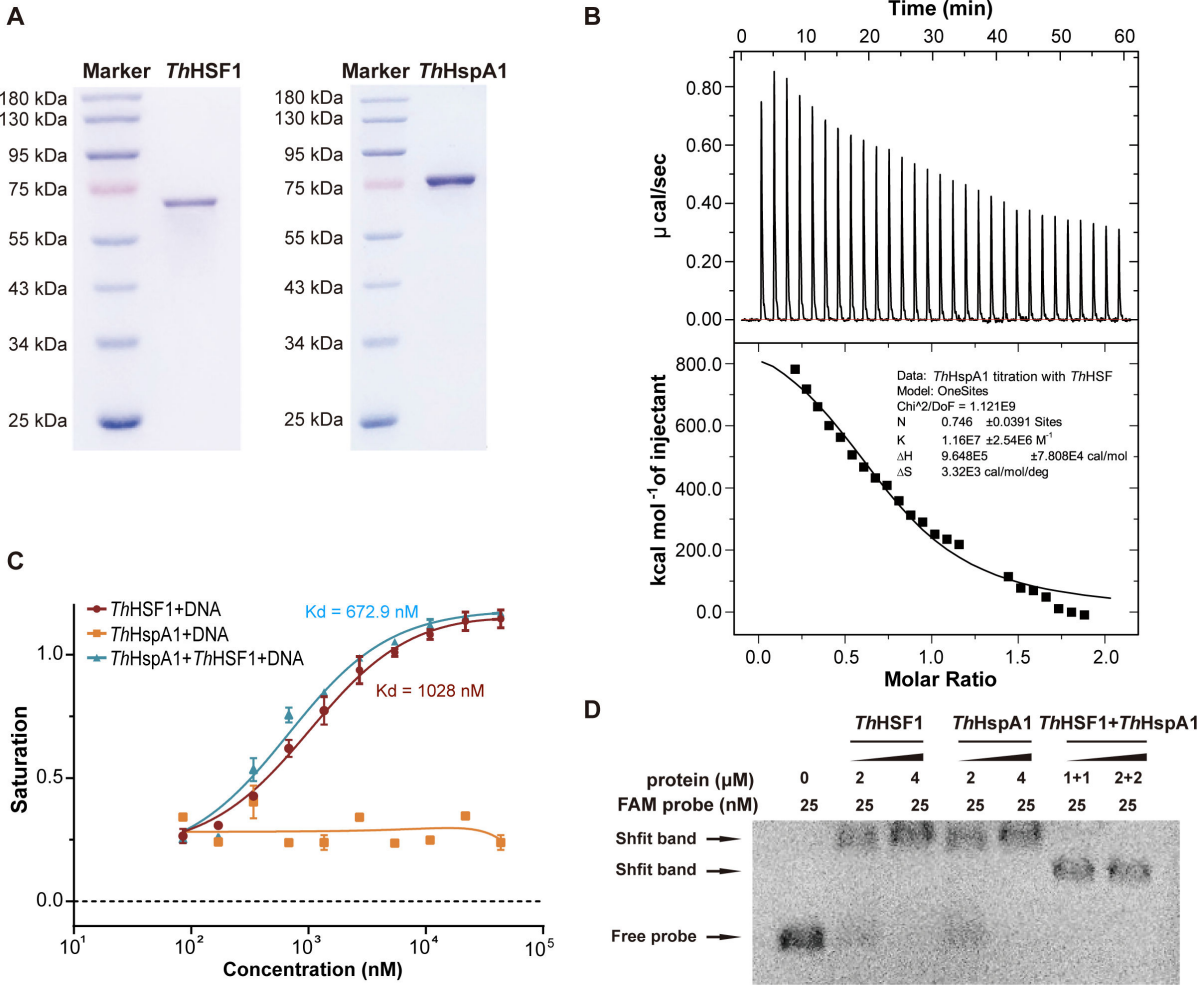
*Th*HSF1 and *Th*HspA1 form a complex to bind to the *lacA* promoter. (**A**) The full-length proteins of *Th*HSF1 and *Th*HspA1 were heterologously expressed in *E. coli* BL21 and purified. (**B**) The ITC experiment between *Th*HspA1 and *Th*HSF1 proteins. (**C, D**) FP (**C**) and EMSA (**D**) assays showed a direct interaction between a mixture of *Th*HSF1 and *Th*HspA1 to the *lacA* promoter (−1,727 to −1,702 bp). The affinity between protein and DNA probe is represented by Kd. Experiments were repeated at least three times, and representative results are shown.

The full-length proteins of *Th*HSF1 and *Th*HspA1 were purified (Fig. 7A), and ITC experiments were conducted to verify their potential interaction. As shown in Fig. 7B, a robust interaction was observed between *Th*HSF1 and *Th*HspA1, with a Kd value of approximately 105 nM. Dynamic light scattering (DLS) analysis indicated the formation of a complex between *Th*HSF1 and *Th*HspA1, whereas *Th*HSF1 alone existed as a trimer or tetramer, and *Th*HspA1 alone presented as a hexamer (41) (Fig. S10). The *lacA* promoter was further selected to investigate the interactions between these two proteins and DNA. FP assays demonstrated the cooperative binding of *Th*HSF1 and *Th*HspA1 to the *lacA* promoter (Fig. 7C). *Th*HspA1 alone did not bind to the DNA probe (−1,727 to −1,702 bp), while the addition of *Th*HspA1 facilitated the formation of a DNA-protein complex with full-length *Th*HSF1, reducing the Kd from about 1,028 nM to 672.9 nM (Fig. 7C). Subsequently, an amplified DNA probe (−1,727 to −205 bp) was utilized in EMSA experiments due to the binding capability of *Th*HspA1 with the DNA fragment spanning from −551 to −205 bp within the *lacA* promoter region (41). Each protein could independently bind to this extended probe (Fig. 7D). Interestingly, there was a noticeable increase in the formation of the DNA-protein complex upon the addition of both proteins, accompanied by a faster migration rate.

**Fig 8:**
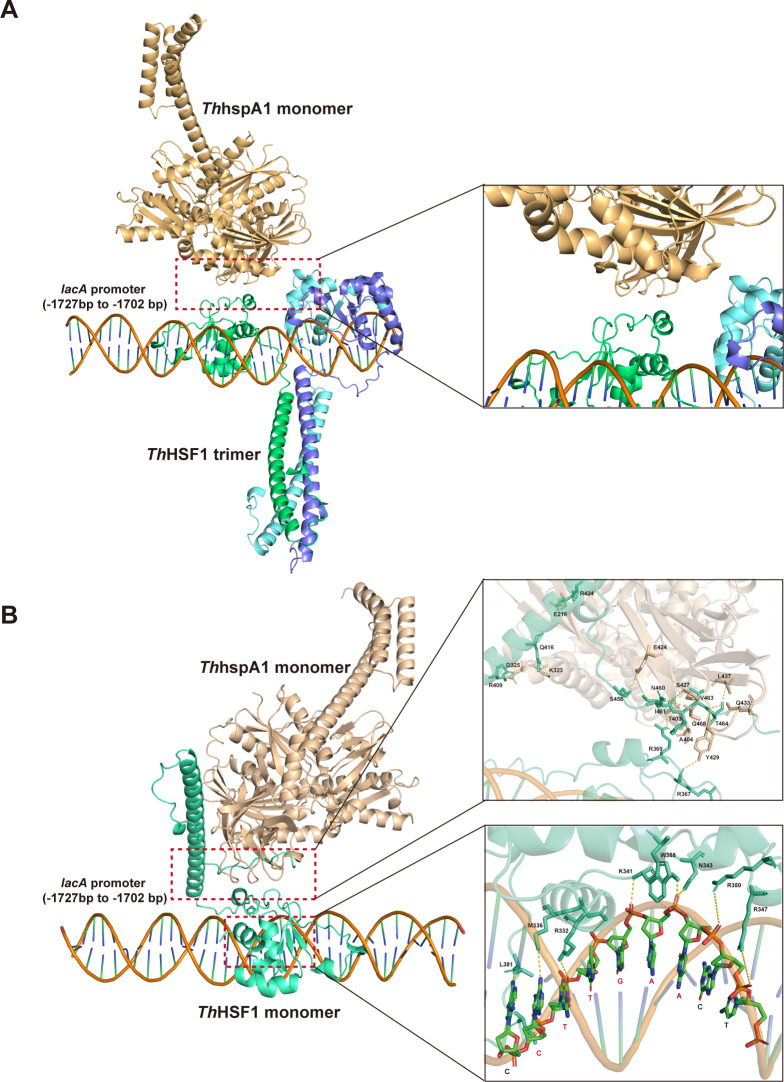
The 3D models of trimeric (**A**) or monomeric (**B**) *Th*HSF1 in complex with *Th*HspA1 and the DNA probe. The DNA probe was derived from the *lacA* promoter (−1,727 to −1,702 bp). A 1:1 *Th*HSF1–*Th*HspA1 complex was used in **B**.

The 3D models of trimeric or monomeric *Th*HSF1 in complex with *Th*HspA1 and the DNA probe derived from the *lacA* promoter (−1,727 to −1,702 bp) were generated using AlphaFold 3 to predict the interaction patterns. As shown in Fig. 8, trimeric *Th*HSF1 bound to the DNA probe without interacting with *Th*HspA1. In contrast, monomeric *Th*HSF1 formed a heterodimeric complex with *Th*HspA1 to interact with the DNA fragment, with *Th*HSF1 mediating direct binding, consistent with the EMSA results (Fig. 7C). The DNA sequence specifically recognized by *Th*HSF1 contained the core conserved motif CTTGAA. In addition, the residues in *Th*HSF1 bound to DNA included R332, M336, K341, N343, R347, R350, W358, and L381. Overall, these results suggested that *Th*HSF1 and *Th*HspA1 exhibited a synergistic effect, forming a regulatory complex to participate in the transcription of LacA in *T. hirsuta* AH28-2 under Cu^2+^ induction.

## DISCUSSION

The laccase gene family in white-rot fungi typically encodes multiple laccase isozymes, exhibiting notably distinct expression patterns under various environmental pressures (50). Cu^2+^, as an essential micronutrient, plays a crucial role in numerous physiological processes across cell types (51). Increased laccase expression associated with intracellular Cu^2+^ accumulation has been observed in many white-rot fungi (24, 36). However, the mechanisms by which fungi respond to Cu^2+^ stress and transmit intracellular signals to initiate differential expression of laccase isozymes remain unclear. This study revealed a Cu²^+^-responsive regulatory mechanism underlying differential laccase isozyme expression in *T. hirsuta*.

According to the native-PAGE analysis (Fig. 4B; Fig. S1), laccase isozyme LacF, along with LacA and LacB, was induced for overexpression following copper exposure. Furthermore, unlike the gradually increasing expression patterns of LacA and LacB in response to Cu^2+^ concentration, the expression level of LacC reached its peak at 100 µM Cu^2+^ and remained stable, while that of LacF peaked at 100–200 µM Cu^2+^ before declining (Fig. S1). To further explore the regulatory basis of these differences, we first examined the global transcriptomic and proteomic responses of *T. hirsuta* to Cu²^+^ stress. Our comparative omics analysis covered 1,682 DEGs and 1,128 DEPs, but only 231 showed consistent changes at both transcript and protein levels (Fig. 1B). This limited overlap indicates a generally weak correlation between the transcriptome and proteome, a pattern commonly observed in fungi and other eukaryotes (52, 53). Such divergence reflects multiple layers of regulation beyond transcription, including differences in mRNA stability, translational efficiency, protein turnover, and stress-induced post-translational modifications. In *T. hirsuta*, copper exposure may further amplify these discrepancies by selectively enhancing the synthesis or stability of proteins involved in oxidative stress defense, metal binding, and molecular chaperones, which are not necessarily mirrored at the transcriptional level. These findings highlight the importance of integrating transcriptomic and proteomic analyses to fully understand fungal responses to metal stress.

HSF is activated when cells are confronted with some environmental stressors, regulating the expressions of various antioxidant stress genes to protect cells from oxidative damage (54). In *T. trogii* and *P. sojae*, HSF is a confirmed key regulator of laccase expression, with overexpression or silencing of *TtHSF2* and *PsHSF1* resulting in varying effects on laccase production (29, 36). Here, we identified a nuclear-localized homologous protein of HSF in *T. hirsuta* AH28-2, sharing up to 45% identity in the DBD region with HSFs from different species (Fig. 2). According to the *in vivo* and *in vitro* experiments, *Th*HSF1 functioned as a Cu^2+^-responsive transcription factor regulating the expressions of laccase isozymes LacA, LacB, and LacF, but not LacC. Although LacC responded to Cu^2+^ stress, its regulation might be mediated by other transcription factors rather than HSF. In fact, multiple intricate transcriptional regulators within cells control the expression of genes involved in stress response (45, 55, 56). For instance, many fungi harbor three orthologs containing HSF domains, namely, Hsf1, Sfl1, and Skn7. These proteins exhibit overlapping or opposing roles in activating various families of *hsp* genes and mediating the differential expression of genes required for development and intracellular homeostasis (57).

HSF typically functions by binding to the HSE element located in the promoters of target genes (58). In eukaryotes, the typical HSE comprises conserved repeats of nGAAn pentanucleotides, which are classified into four types based on the number of repeats and the spacing between them: 4P-type, 3P-type, Gap-type, and Step-type (33). However, the binding sequence in the *lacA* promoter region deviated from these typical HSF binding models and instead revealed a novel hexanucleotide sequence, CTTGAA. Mutation assays through EMSA and FP experiments demonstrated that single-point or complete mutations in either GAA or CTT significantly weakened or abolished *Th*HSF1’s binding to the probes (Fig. 5H and I; Fig. S8). Conversely, *Th*HSF1 bound to the *lacF* promoter in a manner similar to the Gap-type model. This might also account for the inability of *Th*HSF1 to bind to the *lacC* promoter as no matching sequences for any scenario were identified within *lacC*’s 2,000-bp promoter region. Interestingly, most hypothesized HSE elements found upstream of laccase genes in white-rot fungi do not conform to any of these four typical types (11). Notably though, the HSE element in the laccase gene promoter of *P. ostreatus* resembles the binding motif CTTGAA that we discovered here (59).

The relationship between HSF and Hsp70 proteins follows a classic upstream-downstream pattern, with HSF initiating the expression of Hsp70. Moreover, the interaction mechanism between these two proteins has also been gradually elucidated (35, 60, 61). In various species, including *Arabidopsis*, humans, and yeast, Hsp70 acts as a negative feedback regulator by interacting with HSF to inhibit its activity (60, 61). Furthermore, the tight regulation of HSF transcriptional activity is crucial for cellular fitness and maintaining proteostasis. This was demonstrated in yeast, where global dysregulation of *hsf1* transcriptional activity occurred upon the removal of Hsp70 regulation (62, 63). In *T. hirsuta* AH28-2, we confirmed that *Th*HSF1 and the Hsp70 homolog *Th*HspA1 could form a regulatory complex involved in the differential transcription of laccase isozymes. *Th*HspA1 might function as a co-activator as it was capable of binding to the *lacA* promoter alone; however, when mixed with *Th*HSF1 at a 1:1 molar ratio and incubated with the DNA, it did not participate in direct DNA interaction. The 3D modeling of the *lacA* promoter DNA fragment (−1,727 to −205 bp) in complex with *Th*HSF1 and *Th*HspA1 generated similar results (Fig. S11). Nevertheless, the precise assembly architecture of the heterocomplex remained uncertain due to the undefined stoichiometry ratio of the two proteins *in vivo*, which warrants future investigation. The synergistic effects of *Th*HSF1 and *Th*HspA1 resembled the coregulation of HSF and Ssa1 observed in *C. neoformans* (35). These results also explained our previous finding that sole overexpression of *Th*HspA1 led to no significant enhancement in LacA production (41). Additionally, considering that Hsp70 plays a crucial role as a molecular chaperone in facilitating peptide folding, refolding damaged protein, and responding to stress within cells (64, 65), it is possible that post-translational activities executed by *Th*HspA1 are involved in regulating laccase expression.

In summary, under Cu^2+^ stress, four upregulated laccase isozymes were observed in *T. hirsuta* AH28-2, along with the discovery of a Cu^2+^-responsive transcription factor, *Th*HSF1. *Th*HSF1 is selectively bound to the upstream promoter regions of the laccase gene family through specific sequences, thereby differentially regulating the expressions of laccase isozymes in response to copper stress. Furthermore, *Th*HspA1 cooperated with *Th*HSF1 to function in the differential regulation of laccase transcription, providing an efficient strategy to enhance laccase production through strain engineering.

## MATERIALS AND METHODS

### Fungal strains and cultivation conditions

*T. hirsuta* AH28-2 (CCTCC No. AF 2015027, China Center for Type Culture Collection) was sustained on compound potato dextrose agar (CPDA) plates as previously reported (25). The XH medium, with the following composition per liter—15 g cellobiose, 1 g peptone, 1.5 g DL-asparagine, 0.1 g Na_2_HPO_4_, 1 g KH_2_PO_4_, 0.5 g MgSO_4_∙7H_2_O, 0.01 g CaCl_2_, 1 mg FeSO_4_∙7H_2_O, 28 mg adenine, 0.05 mg vitamin B1, and 2 mg CuSO_4_∙7H_2_O—was employed for its liquid cultivation (25). CuSO_4_ was added as necessary at a final concentration of 50, 100, 200, 250, 500, or 1,000 μM.

### Liquid cultivation

Six actively growing blocks of *T. hirsuta* AH28-2 (5 mm in diameter) from the CPDA plates were inoculated into liquid XH medium and maintained with continuous shaking at 120 rpm in the dark for a duration of 96 h. Following homogenization, the primary cultures (5%, vol/vol) were transferred into 100 mL of freshly prepared XH medium. Subsequently, they were pre-cultured with agitation at 120 rpm for 72 h before the addition of different concentrations of CuSO_4_ (41). The addition of CuSO_4_ was designated as the starting point (0 h) for induction. Control cultures were set up without the addition of extra agents. The experiments were conducted thrice, each time with triplicate cultures per test case.

One batch of fresh mycelial cultures of the *T. hirsuta* AH28-2 WT strain exposed to 100 µM Cu²^+^ was randomly selected, and three independent biological replicates were harvested. Each replicate culture was divided into two aliquots: one aliquot was immediately frozen in liquid nitrogen for total RNA extraction and transcriptomic analysis, and the other was subjected to protein extraction and proteomic analysis.

### Transcriptomic analysis

Total RNA was extracted using the TRIzol reagent kit (Tiangen Biotech, Beijing, China). RNA quality and concentration were assessed with a NanoDrop 2000 (NanoDrop Technologies, Inc., Wilmington, DE, USA) and through 1% agarose gel electrophoresis analyzed by the Agilent 2100 Bioanalyzer (Agilent Technologies, Santa Clara, CA, USA). RNA libraries were prepared with poly(A)-selection, cDNA synthesis, adenylation of 3' ends, adapter ligation, DNA fragment enrichment, and purification, followed by sequencing on the Illumina HiSeq 2500 platform at Novogene Bioinformatics Technology Co., Ltd (Beijing, China).

Low-quality reads (quality value ≤20), adapter contamination, and high unknown base content were removed before *de novo* transcriptome assembly with Trinity (66). Gene expression levels were estimated using HTSeq v0.6.0 and Reads Per Kilobase Million Mapped Reads (RPKM) (67). Differential gene expression was analyzed with DESeq2, identifying genes with an FDR below 0.05, and an absolute fold change ≥1 as DEGs (68). The GO enrichment analysis of DEGs utilized the hypergeometric test, considering a q value < 0.05 as significant. DEGs were annotated in the KEGG database for pathway enrichment analysis, with significantly enriched pathways identified based on a corrected P-value (Q-value) ≤0.05 (69).

### Proteomic analysis

Mycelia were collected and lysed with SDT buffer (4% SDS, 100 mM Tris-HCl, 1 mM DTT, pH 7.6). Mycelia without CuSO_4_ were used as the control. The ultrafiltration process was performed using 10 kDa tubes with UA buffer (8 M urea, 150 mM Tris-HCl, pH 8.0) to remove detergent and small molecules effectively. Cysteine residues were alkylated by iodoacetamide in UA buffer, followed by incubation in the dark. The filters were then washed both with UA buffer and NH_4_HCO_3_ buffer. Protein concentration was determined using the BCA assay. For protein digestion, trypsin was used following the filter-aided sample preparation (FASP) protocol (70). Peptides were desalted using C18 cartridges (Empore SPE Cartridges C18, Sigma, USA), concentrated, reconstituted in formic acid, and assessed at a wavelength of 280 nm. Finally, the peptides were separated by SDS-PAGE and visualized with Coomassie Blue.

Samples were analyzed on an EASY-Spray C18 LC column (Thermo Fisher Scientific) and Q Exactive mass spectrometer (Thermo Fisher Scientific). Each sample (10 µL) was separated using a gradient of mobile phases A (water and formic acid) and B (acetonitrile and formic acid) over 126 min. Protein identification and quantification were performed using MaxQuant software (version 1.5.3.17). DEPs were identified with Fisher’s exact test (*P*-value < 0.05, fold change >2.0 or <0.5). GO annotations were predicted using Blast2GO (https://www.blast2go.com/), biological pathways using the KEGG database (http://www.genome.jp/kegg/), and TFs by comparing UniProt IDs with Fungal TFDB1.2 (http://ftfd.snu.ac.kr/index.php?a=view).

### Overlap statistics

Genes and proteins were mapped via one-to-one gene identifiers. Differential features were combined to identify gene–protein pairs with differential regulation in either direction. The expected overlap under independence was computed as μ = n_DEG × n_DEP / N, where N is the total number of detected transcripts (*N* = 10,984 in this study). Significance was evaluated using a hypergeometric test (two-sided) with a confirmatory Fisher’s exact test. Direction-specific counts (up/up, down/down, up/down, and down/up) are shown in Table S1.

### Total laccase enzymatic activity assay and native-PAGE analysis

Laccase activity was assessed by employing guaiacol as the substrate, according to 71. Standard protocol was followed for the analysis of native-PAGE on 10% polyacrylamide gels. The gels were submerged in a solution containing 100 mM citrate-phosphate buffer (pH 4.0) and 15 mM 2,2-azino-bis(3-ethylbenzothiazoline-6-sulfonate) (ABTS) at 25°C for approximately 0.5 h, as described in 72.

### qRT-PCR analysis

*T. hirsuta* AH28-2 strains were cultured continuously in liquid XH media for 0, 24, and 48 h to harvest the mycelia. Subsequently, cDNA synthesis was performed using 1 μg of total RNA as the template, following the protocol provided with the PrimeScript RT kit (Takara). The transcriptional levels of target genes (*Thhsf1*, *ThhspA1*, *lacA*, *lacB*, *lacC*, and *lacF*) were assessed using qRT-PCR with a SYBR green kit (TaKaRa) on a LightCycler 96 real-time PCR system (Roche, Basel, Switzerland). The specific primers for the above target genes are shown in Table S2. The glyceraldehyde-3-phosphate dehydrogenase (*gapdh*) gene served as a constitutively expressed endogenous control (25), and the 2^-ΔΔCT^ method was applied to calculate the relative expression level (73).

### Construction of plasmids and transformation of *T. hirsuta* AH28-2

The construction of the *Th*HSF1 silencing vector pYSK7-an*Thhsf1* (Fig. S6) and overexpression vectors pYSK7-*gfp-Thhsf1* and pYSK7-*Thhsf1* followed the established protocol using the recombinant plasmid pYSK7, as detailed described by Liu et al (74). The amplification of a 251-bp antisense fragment of *Thhsf1* cDNA (from 531 to 781 bp) and a 2,103-bp full-length cDNA of *Thhsf1* was performed using the primer pairs listed in Table S2. Moreover, through overlapping extension PCR and linkage with a linker, a segment of the 2,781-\bp *gfp-Thhsf1* sequence was successfully amplified for the construction of the pYSK7-*gfp-Thhsf1* vector. Either fragment was integrated into the pYSK7 plasmid downstream of the *Agaricus bisporus gpdII* promoter via homologous recombination in *S. cerevisiae* Y1H.

The spores of *T. hirsuta* AH28-2 were harvested, subjected to enzymatic treatment to generate protoplasts, and co-transformed with either of the above three vectors along with the plasmid pCRII-*hph* using the PEG/CaCl_2_ method (41). For *Th*HspA1 and *Th*HSF1 co-overexpression, two vectors, including pYSK7-*Thhsf1* and pCRII-*hph-ThhspA1* (41), were co-transformed. The positive transformants were screened through resistance to hygromycin B, followed by genomic PCR amplification using primers L22 and L24 (Table S2). Two or three gene-silenced or overexpressed transformants were randomly selected, and their transcription levels of *Thhsf1*, *lacA*, *lacB*, *lacC*, and *lacF* were determined by qRT-PCR. Simultaneously, the laccase activity and growth phenotype were also analyzed.

### Localization of *Th*HSF1 in*T. hirsuta* AH28-2

The mycelia of the positive *gfp-Thhsf1*-overexpressed transformant were inoculated into an XH solid medium with or without 100 µM CuSO_4_ addition. Following that, the hyphae were treated with cold methanol, stained with 2 mg/mL DAPI for 5 min in darkness, and images were captured using a laser confocal microscope (Olympus, Japan). The experiments were replicated at least three times.

### Verification of the DNA-binding capacity and transcriptional activation activity of *Th*HSF1 in *S. cerevisiae*

A DNA bait fragment spanning the *lacA* promoter region (−1,800 to −1,650 bp) was amplified using the primer pair Bait-F and Bait-R (Table S2) and cloned into the pAbAi vector (Clontech), which carries the AbA resistance gene *AUR1-C*, via *Sac*I and *Sal*I restriction sites to generate the reporter plasmid pAbAi-Bait. This plasmid was subsequently integrated into the *S. cerevisiae* Y1H genome. As a negative control, the pAbAi plasmid (Takara) was independently incorporated into the Y1H genome to construct the Y1H-pAbAi strain. The pGADT7-*Th*HSF1 plasmid, containing the full-length cDNA of *Th*HSF1, was constructed and co-transformed into both the Y1H-Bait reporter strain and the Y1H-pAbAi control strain. Additionally, pGADT7-RecA (Takara) was used as a positive control. Transformation was serially diluted, titrated (5 µL) on SD/-Ura/-Leu or SD/-Ura/-Leu/AbA^500^ plates, and incubated at 30°C for 3 d (25). A null control was included by transforming the empty pGADT7 vector into the Y1H-Bait reporter strain.

The full-length *lacA* promoter was amplified using the primer pair PlacA-F and PlacA-R (Table S2) and inserted upstream of the *Escherichia coli lacZ* coding sequence in the modified pRS414 plasmid (48) via fusion extension PCR to generate the plasmid pRS414-*P_lacA_*. This plasmid was co-transformed with the above pGADT7-*Th*HSF1 plasmid into yeast strain Y187 to validate the transcriptional activation activity of *Th*HSF1. As a negative control, the empty pGADT7 vector was co-transformed with pRS414-*P_lacA_* under the same conditions. LacZ activity of the transformants was determined by measuring the amount of p-nitrophenol released from the substrate *p*-nitrophenyl-*β*-D-cellobioside (pNPG; Merck).

### Mycelial morphology and growth inhibition rate assay

The WT strain of *T. hirsuta* AH28-2 and three positive *Thhsf1* silencing transformants were inoculated on CPDA solid medium and cultured in the dark at the optimal temperature of 28°C with or without 100 µM CuSO_4_ addition or under elevated temperature conditions at 37°C for 7 d. Subsequently, all colonies from three parallel agar plates were photographed, and the hyphae of *T. hirsuta* AH28-2 were then observed under a ZEISS AXIO Scope A1 microscope (ZEISS, Oberkochen, Germany). Images were captured with an Axiocam 506 color digital camera (Zeiss) under bright-field illumination and processed with Adobe Photoshop 7.0 software (Adobe Inc., San Jose, CA).

### Molecular docking simulations

The full-length and DBD of *Th*HSF1 were individually docked with the promoters of *lacA*, *lacB*, *lacC*, or *lacF* using the HDOCK Server website (http://hdock.phys.hust.edu.cn/). This docking simulation utilized a hybrid algorithm combining template-based modeling and *ab initio* free docking (75). Protein 3D structures were modeled using AlphaFold 3 via the AlphaFold server: (i) trimeric *Th*HSF1 bound to the DNA fragment of the *lacA* promoter (−1,727 to −1,702 bp), with *Th*HspA1 included to assess steric compatibility, and (ii) a 1:1 *Th*HSF1–*Th*HspA1 complex bound to the same DNA fragment. The results were visualized using the Pymol software (version 2.5.2). These fragments were employed for probe synthesis for *in vitro* experimental validations.

### Heterologous expression and purification of full-length *Th*HSF1 and*Th*HSF1 DBD

*Thhsf1* full-length cDNA and *Thhsf1* DBD cDNA fragments were amplified with specific primers (Table S2) and inserted into the pET28a (+) and pET28a-SUMO (+) vectors, generating pET28a-*Thhsf1* and pET28a-SUMO-*Thhsf1*-DBD constructs, respectively. Both of the recombinant plasmids were transformed into *E. coli* BL21 (DE3) for protein expression. Following the induction with isopropyl-β-d-thiogalactopyranoside (IPTG) at a final concentration of 1 mM, the engineered cells were incubated at 16°C for 20—22 h. The cells were lysed using sonication in a cold Tris buffer (40 mM, pH 7.5) supplemented with 500 mM NaCl. The purified recombinant proteins were obtained via affinity chromatography on Ni-NTA Resin (Novagen, Darmstadt, Germany). The SUMO tag was removed through enzymatic cleavage using the ULP enzyme (856087). SDS-PAGE and Bradford analysis were performed to assess the purity and the concentration of the protein, respectively. Additionally, the expression and purification of the full-length *Th*HspA1 were referenced from 41.

### EMSA

All DNA probes, including DNA fragments of the *lacA* promoter (–1,656 to –1,631 bp; –1,727 to –205 bp), *lacB* promoter (–1,979 to –1,944 bp), *lacC* promoter (–2,000 to –1,000 bp; –1,000 to –50 bp), *lacF* promoter (–589 to –554 bp), and six mutant DNA fragments of the *lacA* promoter, were synthesized or amplified, and labeled with 6-FAM at the 5' terminus (Sangon Biotech). In a total reaction volume of 20 µL, 100 nM probes were subjected to incubation with varying concentrations of purified *Th*HSF1 DBD in 5 × binding buffer (pH 6.8) containing 25 mM HEPES, 5 mM MgCl_2_, 150 mM KCl, 1 mM DTT, and 5% glycerol (vol/vol) at 16°C for 30 min. Moreover, to validate specific binding, Polyd(I-C) and a 10-fold concentration of unlabeled DNA without the FAM tag were introduced into the reaction system as a nonspecific competitor and a cold competitive probe, respectively. Following incubation at 37°C for 30 min, the mixtures were separated on 6% native-PAGE (25). The 6-FAM-labeled DNA bands were visualized in the gels using a chemiluminescence imager (Smart Chemi 610; Sagecreation, Beijing, China).

### FP assay

Serial dilutions of purified full-length *Th*HSF1, *Th*HSF1 DBD, and full-length *Th*HspA1 were prepared. The reaction mixtures, each comprising 200 µL, consisted of various FAM-labeled DNA fluorescence probes (DNA fragments of *lacA*, *lacB*, *lacF* promoters, and *mutant1*—*mutant6*) at a final concentration of 50 nM, different concentrations of the three proteins, and 5 × binding buffer. Incubation was carried out at 37°C for 30 min. Fluorescence data were collected using a SpectraMax M5 instrument (Molecular Devices; San Jose, USA) with excitation and emission wavelengths of 285 nm and 535 nm, respectively. The dissociation constants (Kd) were calculated as previously reported using GraphPad (41).

### Western blot

Fresh fungal hyphae were harvested and thoroughly ground using liquid nitrogen. Subsequently, 0.1 g of the powder was added with 200 mL of protein extraction buffer (10 mM Tris-HCl, 0.02% NaN_3_, and 0.001% phenylmethylsulfonyl fluoride, pH 8.0), followed by centrifuging to obtain the supernatant for loading (76). Primary antibodies included anti-*Th*HspA1 polyclonal antibody (GeneScript, Nanjing, China; dilution at 1:5,000) and anti-β-actin (Abclonal, Wuhan, China; dilution at 1:20,000). The secondary antibody was horseradish peroxidase (HRP) goat anti-rabbit IgG (H + L) (Abclonal) at a dilution of 1:8,000. In the end, a BeyoECL Plus Kit (P0018S; Beyotime) was employed for autoradiography.

### Isothermal titration calorimetry (ITC) assay

ITC experiments were conducted to investigate the interactions between *Th*HSF1 and *Th*HspA1 using the iTC200 microcalorimetry system (GE Healthcare) at 25°C. These two proteins were diluted in the same Tris buffer (50 mM Tris, 500 mM NaCl, pH 7.5), with a final concentration of 1 µM for *Th*HSF1 and 5 µM for *Th*HspA1. *Th*HspA1 was then titrated against *Th*HSF1. In each titration, an initial injection of 0.5 µL was followed by successive additions of 2 µL, carried out in 5 second intervals with a total duration of 120 s.

### Statistical analyses

GraphPad Prism (v9.0) was used for statistical analysis of experimental data by one-way ANOVA and Student’s *t*-test, and the significance level was set at 0.05 (*P* ≤ 0.05, *P* ≤ 0.01, *P* ≤ 0.001, or *P* ≤ 0.0001) among samples.

## Data Availability

The mass spectrometry proteomics data have been deposited to the ProteomeXchange Consortium (http://proteomecentral.proteomexchange.org) via the iProX partner repository with the data set identifier PXD044843. The transcriptomics data have been deposited to the Sequence Read Archive (SRA) (https://www.ncbi.nlm.nih.gov/sra) with the data set identifier PRJNA1196105.

## SUPPLEMENTAL MATERIAL

Table S1 spectrum.01004-25-s0001.xlsx Overlap of DEGs and DEPs under Cu²⁺ stress with directionality and statistics.

Table S2 and Figures S1 to S11 spectrum.01004-25-s0002.docx Primers used in this study and images of some results.

ASM does not own the copyrights to Supplemental Material that may be linked to, or accessed through, an article. The authors have granted ASM a non-exclusive, world-wide license to publish the Supplemental Material files. Please contact the corresponding author directly for reuse.
