# Supplementary material for: Deciphering the role of ThHSF1 in the differential expression regulation of laccase isozymes in the white-rot fungus Trametes hirsuta
Source: Microbiol Spectr. 2025 Nov 25;14(1):e01004-25. doi: 10.1128/spectrum.01004-25 (PMC12772356; doi:10.1128/spectrum.01004-25)
Supplement: Table S2 and Figures S1 to S11 — Primers used in this study and images of some results. [file spectrum.01004-25-s0002.docx]

**Deciphering the role of *Th*HSF1 in the differential expression regulation of laccase isozymes in the white-rot fungus *Trametes hirsuta***

Kun Wu^1,2,3#^, Rong Zhu^1,2,3#^, Shiwen Zhao^1,2,3^, Chenkai Wang^1,2,3^, Xinlei Zhang^1,2,3^, Shenglong Liu^1,2,3^, Zemin Fang^1,2,3^, Yazhong Xiao^1,2,3^, Juanjuan Liu^1,2,3*^

^1^ School of Life Sciences, Anhui University, 230601 Hefei, Anhui, China

^2^ Anhui Key Laboratory of Biocatalysis and Modern Biomanufacturing, Hefei 230601, Anhui, China

^3^ Anhui Provincial Engineering Technology Research Center of Microorganisms and Biocatalysis, 230601 Hefei, Anhui, China

^#^Kun Wu and Rong Zhu contributed equally to this work. Author order was determined by the type of their contributions.

*Corresponding author

Juanjuan Liu

Phone/Fax: +86-551-63861928; liu_juan825@ahu.edu.cn

**Supplementary Tables**

**Table S2** **Primers used in this study**

| **Name** | **Sequence (5′-3′)** | **Purpose** |
| --- | --- | --- |
| q*lacA*-F | TCCTTCGTGTTGAATGCCGA | qRT-PCR for *lacA* |
| q*lacA*-R | GTTGATACCGCCCGCAAATC | qRT-PCR for *lacA* |
| q*lacB*-F | CGGGCAACTCGTTCCTGTA | qRT-PCR for *lacB* |
| q*lacB*-R | TCATAGACGACGAGCGGAC | qRT-PCR for *lacB* |
| q*lacC*-F | GCTCAAGTCGACCAGCATC | qRT-PCR for *lacC* |
| q*lacC*-R | AGGGACCTGGAAGTCGTAAA | qRT-PCR for *lacC* |
| q*lacF*-F | GTGTCACGCTTCACAGGGT | qRT-PCR for *lacF* |
| q*lacF*-R | CGTACTCCACCATCGAGCT | qRT-PCR for *lacF* |
| q*gapdh*-F | GCCGCTTCAAGGGCAAAGTC | qRT-PCR for *gapdh* |
| q*gapdh*-R | TGTAGTCGGCACCAACGGA | qRT-PCR for *gapdh* |
| q*Thhsf1*-F | CGAACTCGTCGGAGATGTG | qRT-PCR for *Thhsf1* |
| q*Thhsf1*-R | CAAGGTCGAACGGCTGG | qRT-PCR for *Thhsf1* |
| q*ThhspA1*-F | ATCCAGGTCTTCGAGGGTGA | qRT-PCR for *ThhspA1* |
| q*ThhspA1*-R | CGGAGACGTTGAGGATACCG | qRT-PCR for *ThhspA1* |
| *Thhsf1*-anti-F | CTCCCATCTACACACAACAAGCTTATCGCCCGTGAAGCGCGTTGCCT | cloning of *Thhsf1* antisense |
| *Thhsf1*-anti-R | CACTGGCCCTCTGGTCAACTATAATATTATGTCGGAGAAATCCGTCCC | cloning of *Thhsf1* antisense |
| *L22*-*gfp*-F | CTCCCATCTACACACAACAAGCTTATCGCCATGGTGAGCAAGGGCGAGGAG | cloning of *gfp-Thhsf1* for pYSK7-*gfp*-*Thhsf1* |
| *linker*-*gfp*-R | GCTGCCGCTGCCGCTGCCGCTCTTGTACAGCTCGTCCATGC | cloning of *gfp-Thhsf1* for pYSK7-*gfp*-*Thhsf1* |
| *linker*-*Thhsf1*-F | AGCGGCAGCGGCAGCGGCAGCATGTCTTCCCAAGACGGCCCCT | cloning of *gfp-Thhsf1* for pYSK7-*gfp*-*Thhsf1* |
| *L24*-*Thhsf1*-R | CACTGGCCCTCTGGTCAACTATAATATTATTCAGCTCGACGGCTGCTTCG | cloning of *gfp-Thhsf1* for pYSK7-*gfp*-*Thhsf1* |
| *L22-Thhsf1-F* | CACACAACAAGCTTATCGCCATGTCTTCCCAAGACGGCCC | cloning of *Thhsf1* full-length for pYSK7-*Thhsf1* |
| *L24-Thhsf1-R* | GCCCTCTGGTCAACTATAATATTATTCAGCTCGACGGCTGCTTCG | cloning of *Thhsf1* full-length for pYSK7-*Thhsf1* |
| *L22* | ACATCCACCATCTCCGTTTTCTCCCATCTACACACAACAAGCTTATCGCC | genomic PCR amplification |
| *L24* | TGACTATAGCAGCCTCCTACCACTGGCCCTCTGGTCAACTATAATATTAT | genomic PCR amplification |
| *Thhsf1*-pet-F | CGCGGATCCATGTCTTCCCAAGACGGCCCCT | cloning of *Thhsf1* full-length for pET28a-*Thhsf1* |
| *Thhsf1*-pet-R | ATAAGAAGCGGCCGCTCAGCTCGACGGCTGCTTCGTCAT | cloning of *Thhsf1* full-length for pET28a-*Thhsf1* |
| *dbd*-pet-F | CGCGGATCCATGCTCAACAAGCCG  CTGGGG | cloning of *Thhsf1* DBD for pET28a-sumo-DBD |
| *dbd*-pet-R | ATAGTTTAGCGGCCGCTTACTTGAGCGTTGGATCGGGCT | cloning of *Thhsf1* DBD for pET28a-sumo-DBD |
| *ThhspA1*-pet-F | ATATACATATGCACCATCACCACCATCATCACCATACCAAGGCGATCGGTATTGA | cloning of *ThhspA1* full-length for pET22b-*ThhspA1* |
| *ThhspA1*-pet-R | TTCGGATCCTTAGTCGACCTCCTCGACGC | cloning of *ThhspA1* full-length for pET22b-*ThhspA1* |
| *lacC*-probe1-F | CGCTTTCCCTGTTCTACCCTT | Amplification of the *lacC* promoter region probe |
| *lacC*-probe1-R | GAAGACTTTCCCGTCGCTCAG | Amplification of the *lacC* promoter region probe |
| *lacC*-probe2-F | CCCGCATCTGTGCGCTGCGCT | Amplification of the *lacC* promoter region probe |
| *lacC*-probe2-R | TGTTCTCGCGGCAACCAGCCA | Amplification of the *lacC* promoter region probe |
| *HSF1*-*hspA1*-probe-F | GGTGTCAGCATCTGTCAAGTC | Amplification of the *lacA* promoter region probe |
| *HSF1*-*hspA1*-probe-R | TACGCCTTGAACTGACTTACA | Amplification of the *lacA* promoter region probe |
| Bait-F | TGTCACCTTCGGCATCGTCT | Amplification of the bait DNA for the reporter plasmid pAbAi-Bait |
| Bait-R | CTGACAGTCCGGCCAGCGAT | Amplification of the bait DNA for the reporter plasmid pAbAi-Bait |
| PlacA-F | AGATGTTGAGACTGCAGGC | Amplification of the full-length *lacA* promoter |
| PlacA-R | GGCTGTGAAGGGAAGAG | Amplification of the full-length *lacA* promoter |

**Supplementary Figures**


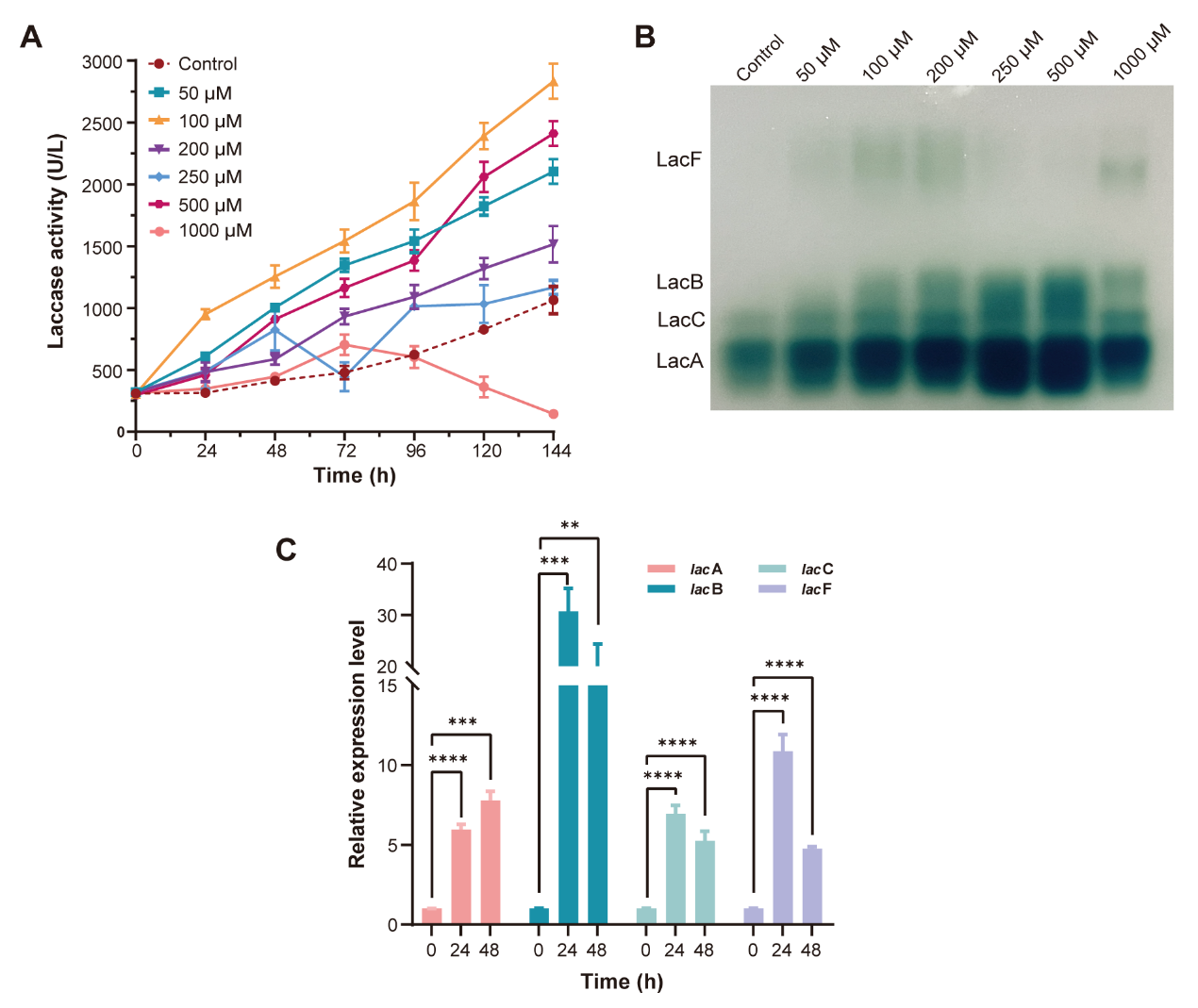


**Figure S1** Expression profile of *T. hirsuta* AH28-2 laccase isozymes upon exposure to different concentrations of Cu^2+^. (A, B) The total laccase activity (A) and isozyme analysis by native-PAGE (B) under Cu^2+^ treatment (0, 50, 100, 200, 250, 500, and 1000 μM). (C) The transcriptional levels of *lacA*, *lacB*, *lacC*, and *lacF* in *T. hirsuta* AH28-2 when exposed to 100 μM Cu^2+^. Data show mean ± SD, n = 3 (***P* < 0.01, ****P* < 0.001, *****P* < 0.0001).


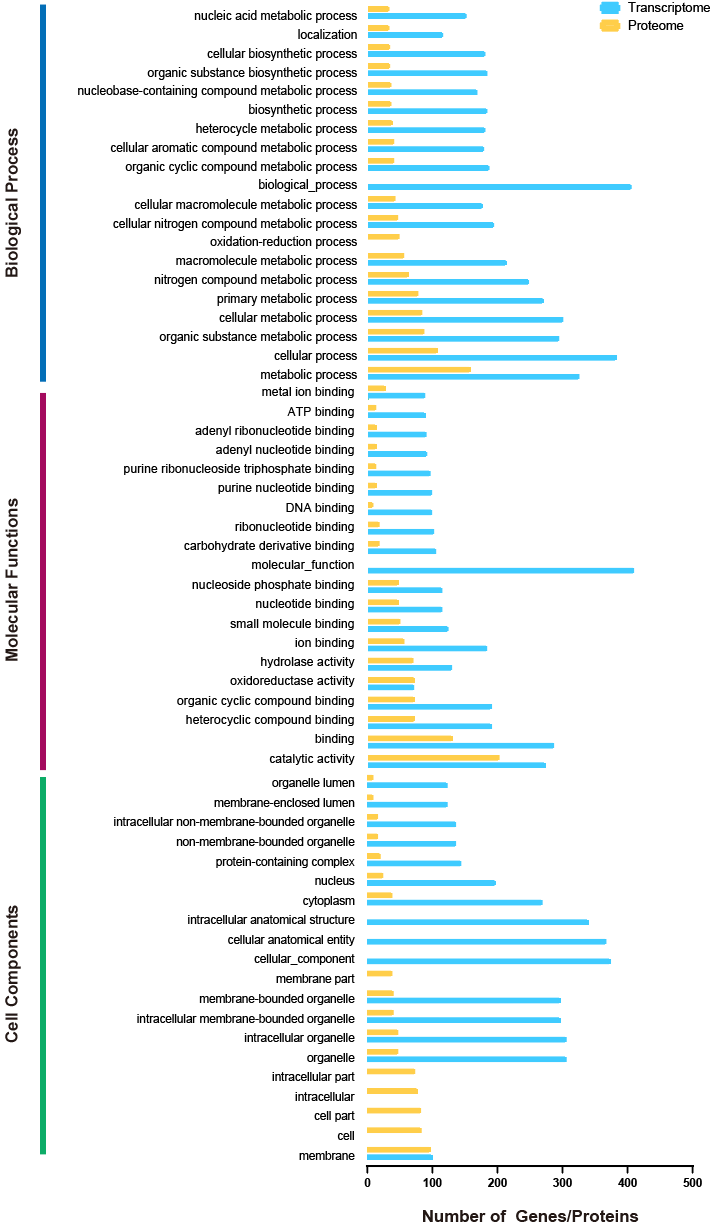


**Figure S2** The GO function terms based on transcriptome and proteome analysis of *T. hirsuta* AH28-2 upon exposure to 100 μM Cu^2＋^.


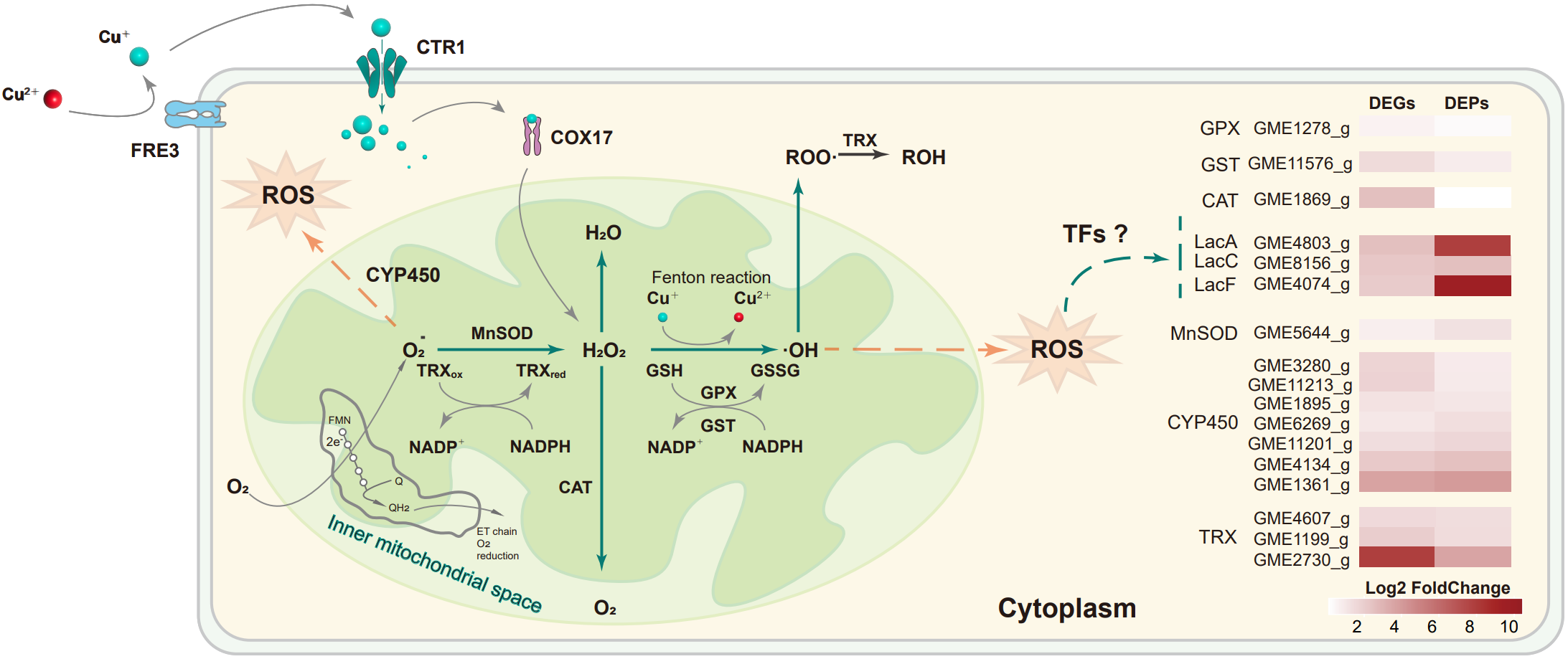


**Figure S3** Cu^2＋^-induced upregulation of antioxidative enzymes based on transcriptome and proteome analysis in *T. hirsuta* AH28-2.


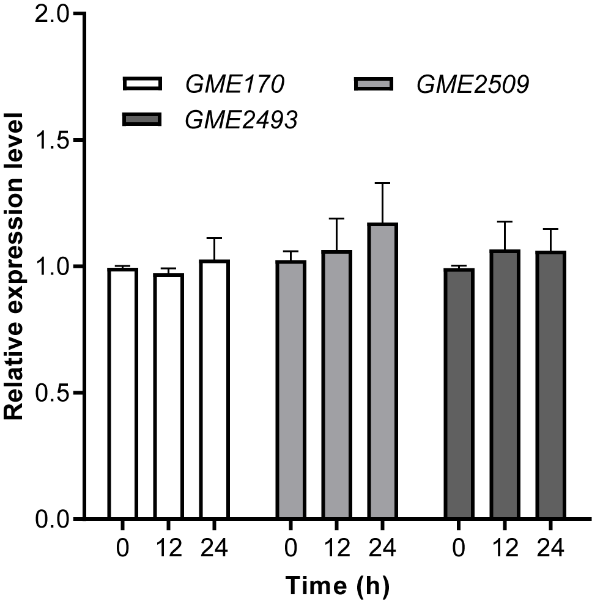


**Figure S4** The transcriptional levels of the other three putative heat shock-type TF genes. Data show mean ± SD.


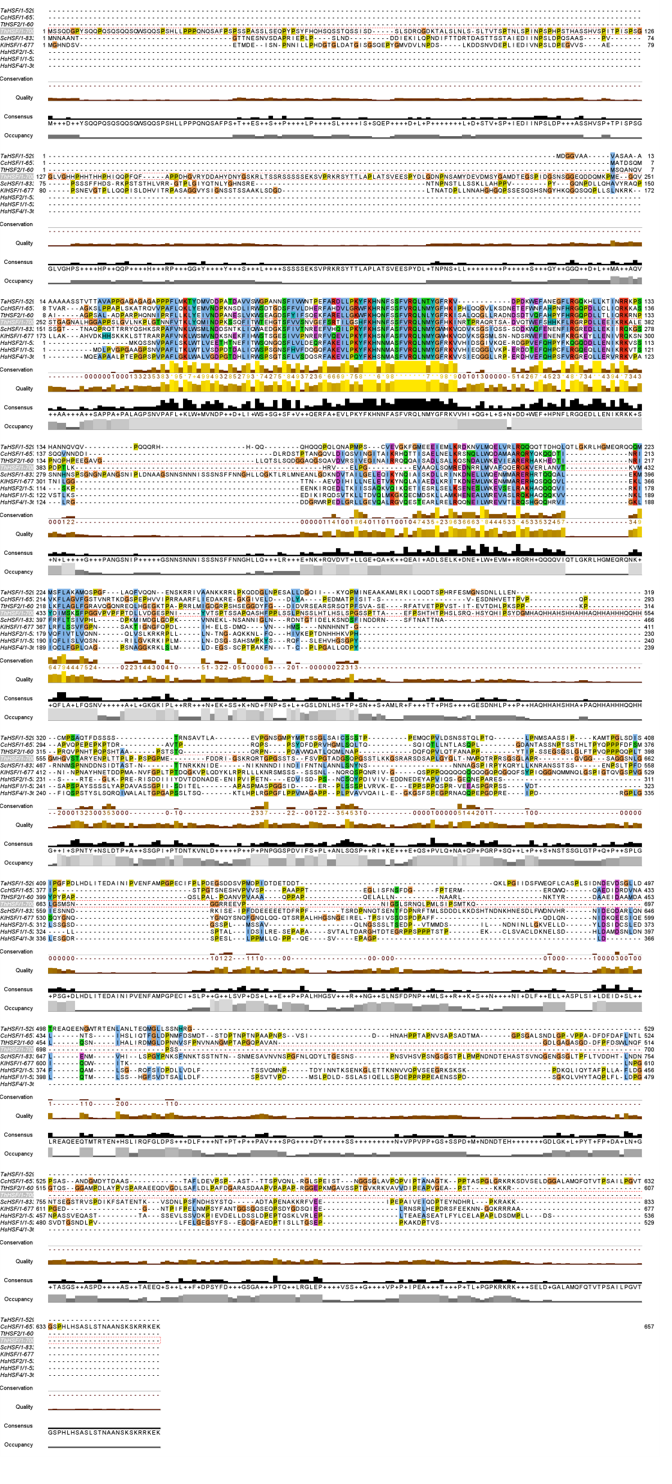


**Figure S5** Amino acid alignment of *Th*HSF1 and its homologs from other species. *Ta*HSF (Gene ID: 123114421) of *T. aestivum L.*, *Cc*HSF (Gene ID: 6017888) of *C. cinerea*, *Tt*HSF2 of *T. trogii*, *Sc*HSF (GenBank: CAA96777.1) of *S. cerevisiae*, *Kl*HSF (GenBank: CAA38950.1) of *Kluyveromyces lactis*, and *Hs*HSF1 (Gene ID: 3297), *Hs*HSF2 (GenBank: AAA36017.1), and *Hs*HSF4 (GenBank: BAA84581.1) of *Homo sapiens* are included.


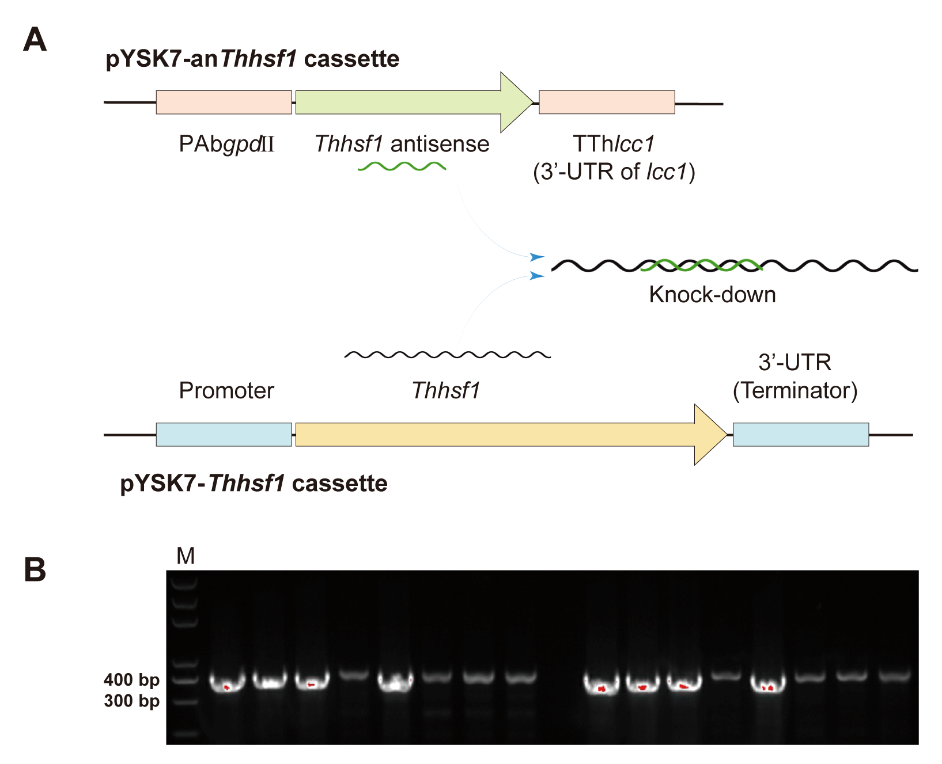


**Figure S6** Schematic representation and experimental verification of the construction of *Thhsf1*-silenced *T. hirsuta* AH28-2. (A) Schematic representation of the pYSK7-an*Thhsf1* cassette used for antisense knock-down. The antisense fragment was integrated into the pYSK7 plasmid downstream of the *Agaricus bisporus* *gpdⅡ* promoter via homologous recombination in *S. cerevisiae* Y1H. (B) Genomic PCR verification of *Thhsf1*-silenced transformants. Lane 1, DNA marker. Lanes 2–18, independent transformants (R*Th*HSF1-20 to R*Th*HSF1-36) showing the expected amplicon with the primer pair targeting the antisense cassette (see Table S2).


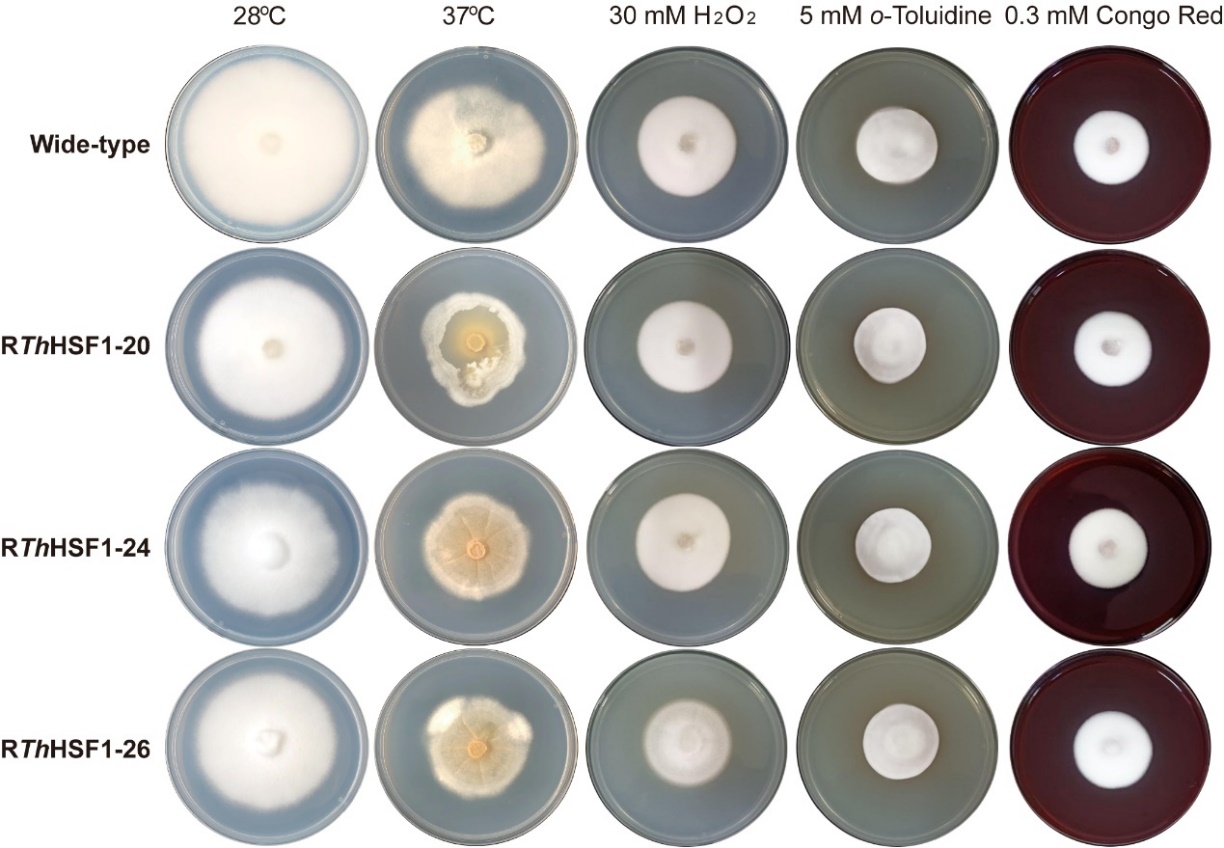


**Figure S7** Mycelial morphology of the WT and *Thhsf1*-silenced *T. hirsuta* AH28-2 on CPDA medium under different conditions. Treatments included normal temperature (28°C, optimal growth condition without any stress), elevated temperature (37°C), oxidative stress (30 mM H₂O₂), aromatic amine stress (5 mM *o*-Toluidine), and cell wall stress (0.3 mM Congo Red). Unless otherwise specified, all cultures were incubated at the optimal growth temperature of 28^o^C.


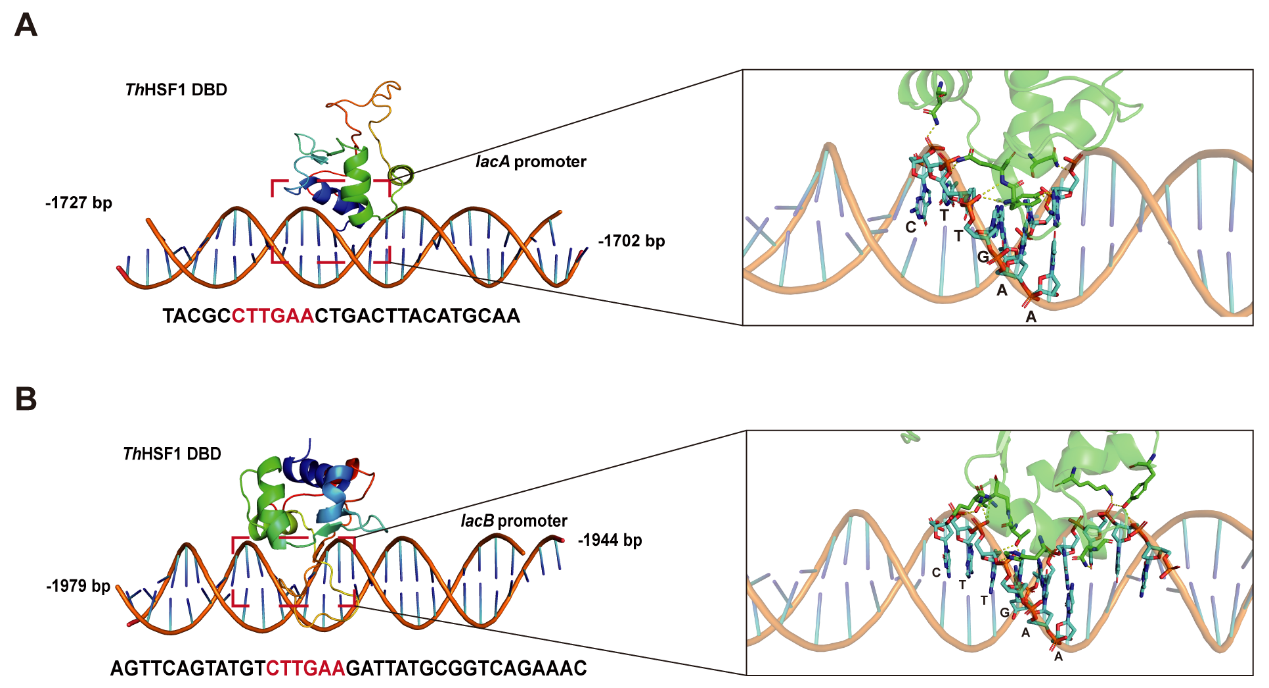


**Figure S8** Prediction of *Th*HSF1 binding sequences to the *lacA* (A) and *lacB* (B) promoter regions using HDOCK.


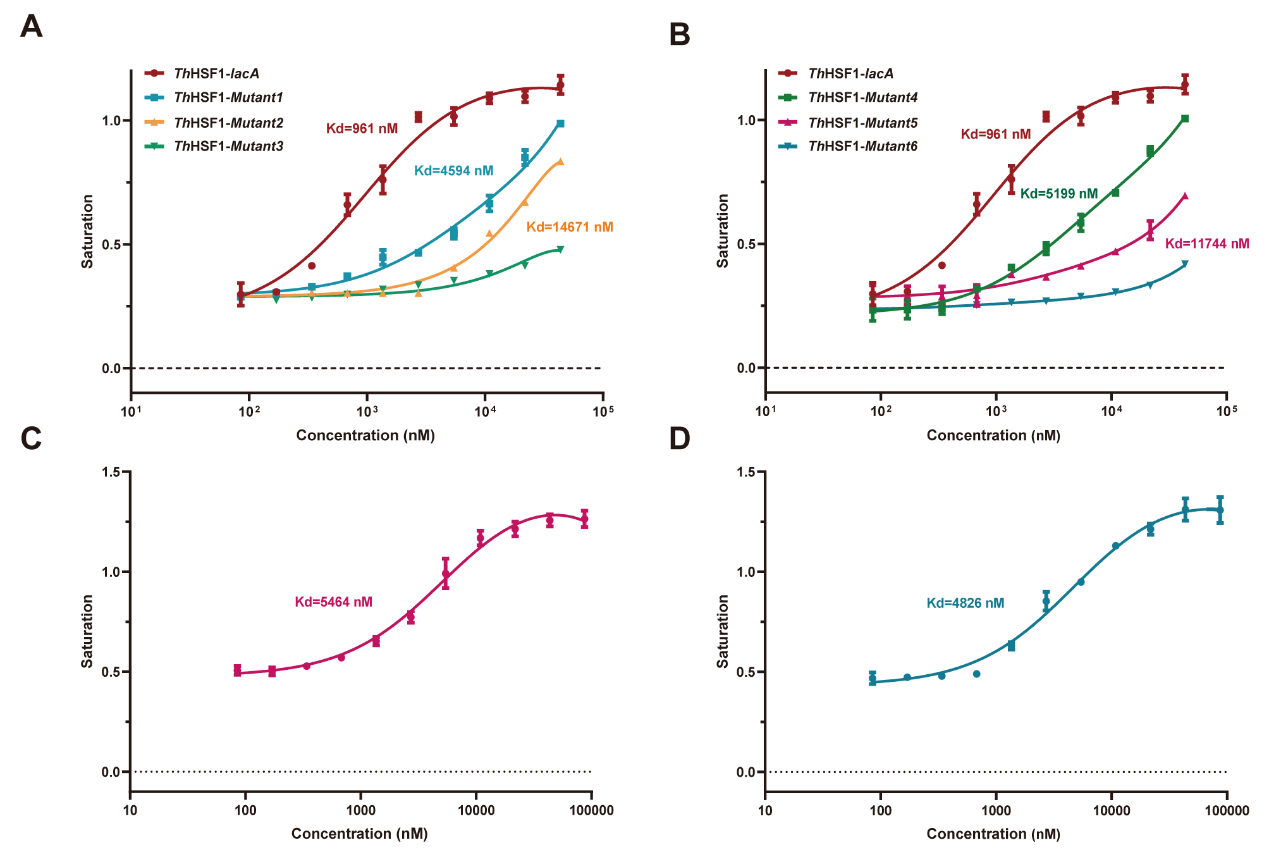


**Figure S9** The dissociation constant (Kd) of *Th*HSF1 and DNA probes detected by FP assays *in vitro*. (A, B) *Th*HSF1 exhibited Kd of 961 nM, 4594 nM, 14671 nM, 5199 nM and 11744 nM with the WT probe of *lacA*, mutant probe 1, mutant probe 2, mutant probe 4, and mutant probe 5, respectively. No complex formation was observed between *Th*HSF1 and mutant probe 3 or mutant probe 6. (C, D) The binding affinities of *Th*HSF1 with *lacB* and *lacF* promoters were characterized by Kd of 5464 nM and 4826 nM, respectively. Each experiment was repeated at least three times and representative results are shown.


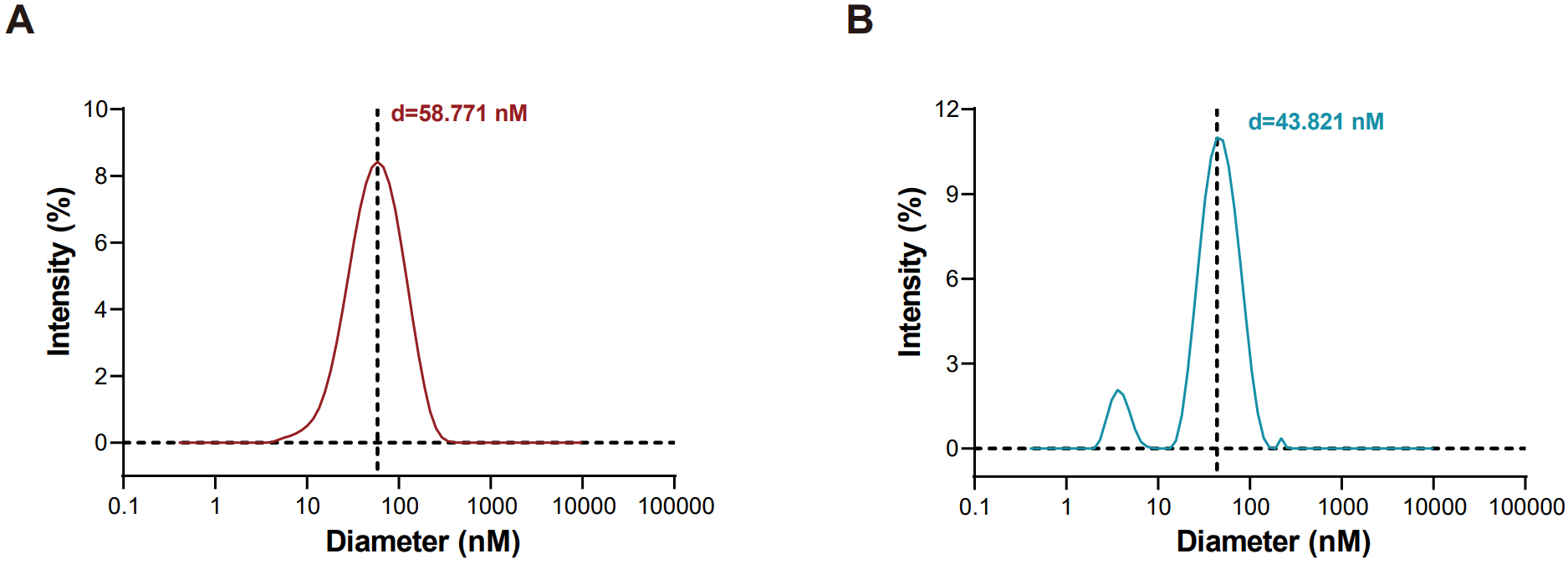


**Figure S10** Measurement of protein diameter by dynamic light scattering experiment. (A) The full-length protein of *Th*HSF1. Particle diameter detection indicated that *Th*HSF1 existed as trimers or higher oligomers. (B) The mixed *Th*HSF1 and *Th*HspA1 protein. Particle diameter detection indicated the formation of a complex between *Th*HSF1 and *Th*HspA1, which may represent a heterodimer of the two proteins.


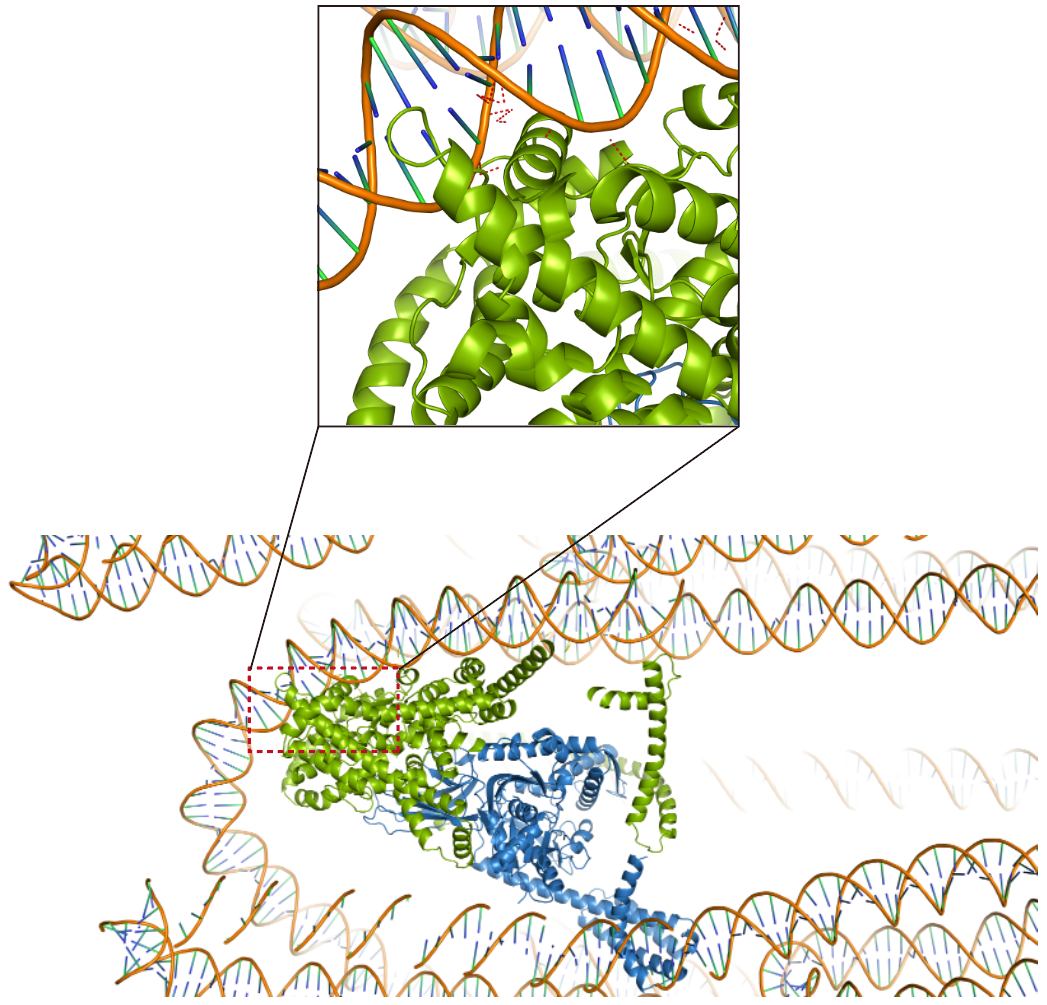


**Figure S11 The 3D models of monomeric *Th*HSF1 in complex with *Th*HspA1 and the long DNA probe.** The DNA probe was derived from the *lacA* promoter (−1727 to −205 bp). A 1:1 *Th*HSF1–*Th*HspA1 complex was used. *Th*HSF1 is shown in green and *Th*HspA1 is shown in blue.
